# Changes in Gene Expression in Leaves of Cacao Genotypes Resistant and Susceptible to *Phytophthora palmivora* Infection

**DOI:** 10.3389/fpls.2021.780805

**Published:** 2022-02-08

**Authors:** Indrani K. Baruah, Shahin S. Ali, Jonathan Shao, David Lary, Bryan A. Bailey

**Affiliations:** ^1^Sustainable Perennial Crops Laboratory, United States Department of Agriculture/Agricultural Research Service, Beltsville Agricultural Research Center-West, Beltsville, MD, United States; ^2^Department of Viticulture and Enology, University of California, Davis, Davis, CA, United States; ^3^United States Department of Agriculture/Agricultural Research Service, Northeast Area, Beltsville, MD, United States; ^4^Department of Physics, University of Texas, Dallas, TX, United States

**Keywords:** cacao, black pod rot, induced defense, RNA-Seq – RNA sequencing, zoospores

## Abstract

Black pod rot, caused by *Phytophthora palmivora*, is a devastating disease of *Theobroma cacao* L. (cacao) leading to huge losses for farmers and limiting chocolate industry supplies. To understand resistance responses of cacao leaves to *P. palmivora*, Stage 2 leaves of genotypes Imperial College Selection 1 (ICS1), Colección Castro Naranjal 51 (CCN51), and Pound7 were inoculated with zoospores and monitored for symptoms up to 48 h. Pound7 consistently showed less necrosis than ICS1 and CCN51 48 h after inoculation. RNA-Seq was carried out on samples 24 h post inoculation. A total of 24,672 expressed cacao genes were identified, and 2,521 transcripts showed induction in at least one *P. palmivora*-treated genotype compared to controls. There were 115 genes induced in the *P. palmivora*-treated samples in all three genotypes. Many of the differentially expressed genes were components of KEGG pathways important in plant defense signal perception (the plant MAPK signaling pathway, plant hormone signal transduction, and plant pathogen interactions), and plant defense metabolite biosynthesis (phenylpropanoid biosynthesis, α-linolenic acid metabolism, ethylene biosynthesis, and terpenoid backbone biosynthesis). A search of putative cacao resistance genes within the cacao transcriptome identified 89 genes with prominent leucine-rich repeat (LRR) domains, 170 protein kinases encoding genes, 210 genes with prominent NB-ARC domains, 305 lectin-related genes, and 97 cysteine-rich RK genes. We further analyzed the cacao leaf transcriptome in detail focusing on gene families-encoding proteins important in signal transduction (MAP kinases and transcription factors) and direct plant defense (Germin-like, ubiquitin-associated, lectin-related, pathogenesis-related, glutathione-S-transferases, and proteases). There was a massive reprogramming of defense gene processes in susceptible cacao leaf tissue after infection, which was restricted in the resistant genotype Pound7. Most genes induced in Pound7 were induced in ICS1/CCN51. The level of induction was not always proportional to the infection level, raising the possibility that genes are responding to infection more strongly in Pound7. There were also defense-associated genes constitutively differentially expressed at higher levels in specific genotypes, possibly providing a prepositioned defense. Many of the defense genes occur in blocks where members are constitutively expressed at different levels, and some members are induced by Ppal infection. With further study, the identified candidate genes and gene blocks may be useful as markers for breeding disease-resistant cacao genotypes against *P. palmivora*.

## Introduction

*Theobroma cacao* L. (cacao), the source of chocolate, is an important cash crop in the tropics with a total harvest of 4.7 million metric tons (ICCO, 2017). Cacao production is threatened by multiple diseases, including black pod rot (BPR) (Marelli et al., 2019). BPR is responsible for more than half the reported crop loss due to disease, destroying the equivalent of 2 billion United States dollars’ worth of dried cacao beans annually (Marelli et al., 2019). BPR is caused by multiple *Phytophthora* species, among which *Phytophthora palmivora* (Ppal) is the most widespread, causing the greatest yield losses on a global scale (Ploetz, 2016).

*Phytophthora palmivora* is a broad host range hemibiotrophic pathogen, and on cacao, it can affect all plant parts (Ali et al., 2016). The Ppal asexual life cycle is characterized by adhesion of mobile zoospores to the host, zoospore encystment, and germ tube formation (Judelson and Blanco, 2005). On cacao, Ppal enters the plant primarily through stomata (Ali et al., 2016) and establishes an apoplastic hyphal network. During the short biotrophic stage, haustoria penetrate cells and release effectors establishing infections (Petre and Kamoun, 2014) and counteracting plant defenses and reprogramming host cells (Morales-Cruz et al., 2020).

Resistance to *Phytophthora* infection is widespread (Thevenin et al., 2012) and quantitatively inherited (Iwaro et al., 2000; Lanaud et al., 2009). Quantitative trait loci (QTLs) for resistance to BPR have been reported (Brown et al., 2007; Lanaud et al., 2009; Akaza et al., 2016; Zhang and Motilal, 2016; Barreto et al., 2018; Mucherino Muñoz et al., 2021). Thirteen consensus QTLs were identified on Chromosomes 1, 2, 4, and 5 from 65 total QTLs spread over all of cacao’s ten chromosomes in an analysis of available QTL data in 2009 (Lanaud et al., 2009). Cacao genotype Pound7 carries resistance to BPR in pods associated with QTLs on Chromosomes 2, 4, 8, and 10 (Brown et al., 2007; Gutiérrez et al., 2021).

Plants have evolved complex systems for detecting microbes involving the binding of pathogen-associated molecular patterns (PAMPs)/damage-associated molecular patterns (DAMPs) to plasma membrane spanning pattern recognition receptors (PRRs), activating immunity responses (Saijo et al., 2018). Once pathogen-triggered immunity (PTI) is activated, defense molecules are delivered to plant-oomycete interfaces, including pathogenesis-related (PR) proteins, callose for thickening cell walls, and antimicrobial toxins. A large family of nucleotide-binding/leucine-rich-repeat NLR receptors have been recruited to detect intracellular interference by diverse pathogen effectors and initiate effector-triggered immunity (ETI) (Cui et al., 2015).

Varying expression levels and gene polymorphisms in PRRs, transcription factors (TFs), and downstream genes are thought to contribute to quantitative resistance in cacao against *Phytophthora* infection (Lanaud et al., 2009). Studies of the cacao response to *Phytophthora megakarya* and *P. palmivora* infection in susceptible cacao pods (Ali et al., 2017) identified differentially expressed cacao genes associated with multiple defense gene classes and pathways. Transcriptomic analysis of the Ppal response in the resistant genotype SCA6 and the susceptible genotype NA32 revealed a transcriptomic response involving PR genes, PRRs, and resistance genes in the resistant genotype (Pokou et al., 2019). In a more recent study of plant-specialized phenylpropanoid metabolites, leaves of Sca6 were found to constitutively accumulate higher levels of clovamide, a hydroxycinnamic acid amide, than the susceptible genotype Imperial College Selection 1 (ICS1), providing a preformed resistance component. Clovamide also inhibits the growth of *Phytophthora* (Knollenberg et al., 2020).

Resistance to BPR in pods causes a research bottleneck due to issues associated with pod availability and difficulties in their manipulations. As a result, leaves have been used as proxies for pods (Iwaro et al., 1997; Tahi et al., 2006), although how the reactions compare remains incompletely understood. The current study uses the oomycete pathogen *P. palmivora* to investigate host defense mechanisms in immature young red leaves (Stage 2) of three cacao genotypes. The cacao genotypes used include the resistant genotype Pound7, the widely planted Colección Castro Naranjal genotype (CCN51), and the susceptible genotype ICS1 (Fister et al., 2020). The objective is to characterize the cacao transcriptome in the resistant/susceptible cacao interaction in Stage 2 cacao leaves, identifying genes of potential importance in the defense response of cacao against *P. palmivora* infection.

## Materials and Methods

### Leaf Sampling

Healthy young red leaves also known as Stage 2 leaves, which are 5- to 10-cm long and pliable (Bailey et al., 2005), were sampled from clonally propagated cacao genotypes Pound7, ICS1, and CCN51 trees in replicates of 4 from separate individual trees of each genotype. The trees were produced by vegetative propagation. Sampling was random based on the availability of flushing Stage 2 leaves on individual trees of each genotype with replications initiated on separate days. These trees are maintained in the USDA-ARS, Beltsville, MD cacao greenhouse at ambient relative humidity and a minimum day length of 12 h using a supplemental light intensity of at least 325 μmoles/m^2^/s.

### *Phytophthora palmivora* Inoculum Preparation

The *Ppal* isolate Gh-ER1349 used was isolated from BPR-infected cacao in Ghana (Ali et al., 2016) and maintained on a clarified V8 juice (CV8) agar plate at 18°C. Zoospore inoculum was produced as described by Lawrence (1978) and modified by Ali et al. (2016). Ppal was grown on a CV8 agar plate (90 mm) for 7 days under constant dark at 25°C and then transferred to constant light (200 lux) for 4–5 days. Plates were flooded with cold sterile water (4°C) and kept at 4°C for 60 min, and then transferred to 28°C for 30 min. The zoospore suspension was transferred to a sterile 50-ml beaker, and the concentration was determined by counting zoospores using a hemocytometer (KOVA Glasstic Slide 10, KOVA International Inc.).

### Leaf Inoculation

Leaves were cut in half across the midvein and transferred abaxial side up to 90-mm Petri dishes containing a wet Whatman No. 2 filter paper. Four zoospore concentrations (6 × 10^5^, 1.5 × 10^5^, 0.6 × 10^5^, and 0.3 × 10^5^ zoospores/ml) were used to titer the response to inoculation. Inoculations were performed by placing 20-μl droplets of zoospore suspension between the minor veins on both the left and right sides of one leaf half. The other leaf half was used as a control and was treated with 20 μl of sterile distilled water. Plates containing inoculated and control leaves were covered with lids, wrapped with parafilm, and transferred to a growth chamber kept at 25°C with 50% relative humidity under 12-h light and dark cycles. Four separate leaves were used for each of four zoospore concentrations for each of three genotypes, and the sample set was duplicated, allowing the harvest of samples at 24-h post inoculation (hpi) and measurement of necrosis at 48 hpi. At 24 hpi, one sample set was harvested and frozen in Liquid N_2_. For the second sample set, each leaf section was scored for the percentage necrotic leaf area at 48 hpi by observing the area under a dissecting microscope at 10× magnification. Controls were asymptomatic; therefore, the percent necrosis data at 48 hpi were analyzed using a one-factor (three genotypes treated with Ppal) random model ANOVA with Tukey’s multiple comparisons test (*p* = 0.05) with four replications for each zoospore dilution.

### RNA Extraction

Total RNA was extracted from flash-frozen-infected and control leaves 24 hpi with the 0.6 × 10^5^ zoospores/ml concentration. Three of four replicates from the pathogen infection assay, chosen at random, were used for RNA-Seq analysis. A total of nine infected (three replicates for three genotypes) and nine control (three replicates for three genotypes) leaf tissue samples were grounded finely in mortar and pestle, and extraction was performed as previously described (Bailey et al., 2013). RNA purity (quality) and concentration (quantity) were assessed using an Axygen Gel Documentation system and a Nanodrop2000 spectrophotometer (Thermo Fischer Scientific, Waltham, MA, United States) and were in the required quality range of the 1.8–2 A_260/280_ absorbance ratio.

### mRNA Isolation, cDNA Synthesis, and Sequencing

Isolation of mRNA, cDNA synthesis, and library assembly and sequencing were outsourced and carried out by Beijing Genomics Institute (BGI), Hong Kong. Initial RNA quality was verified using the RNA integrity number obtained using an Agilent Technologies 2100 bioanalyzer. Libraries were also validated using an Agilent Technologies 2100 bioanalyzer. The final quantified libraries of 18 samples were sequenced on the DNBseq platform to generate paired-end reads of 150 bp and were performed by following methodologies for preparing DNBSeq libraries by BGI, Hong Kong. The libraries were amplified with phi29 creating DNA nanoballs (DNB), and DNBs were loaded into a patterned nanoarray; and single-end 50 (pair end, 100/150) bases reads were generated using combinatorial Probe-Anchor Synthesis (cPAS).

### RNA-Seq Analysis

The sequences acquired by RNA-Seq were verified, in-house, by comparison with the coding sequences (CDS) of the cacao Matina1-6 genome v1.2 (Motamayor et al., 2013). RNA reads from RNA-Seq libraries ranging from 50 to 70 million reads in fastq format were trimmed up using BBDuk version 37.58 (Bushnell, 2014), using adapters.fa with parameters ktrrim = r, k = 23, mink = 11, hist = 1, tpe, tbo. Trimmed reads were aligned using HISAT2 2.1.0 (Pertea et al., 2016) to the cacao CDS (Motamayor et al., 2013). Tabulated raw counts from each CDS were obtained from the HISAT2 alignment. Raw counts were normalized using the DESeq2 (Anders and Huber, 2010) (Galaxy Version 2.11.40.1) available in the Galaxy pipeline.^1^ Trimmed reads were aligned with the CDS of the Ppal isolate Gh-ER1349 genome (Morales-Cruz et al., 2020).

The summed Ppal reads for each library were normalized by comparing the total cacao reads for each library to the average total cacao reads across all libraries and then subtracting the average Ppal reads for controls of each genotype from the Ppal reads of an individually treated sample of each genotype. The resulting numbers for Ppal-treated samples had been log_10_ transformed before analysis across the three genotypes. The Ppal transcript read data at 24 hpi was analyzed using a one-factor (three genotypes treated with Ppal) random model ANOVA with Tukey’s multiple comparisons test (*p* = 0.05) with three replications for each genotype. Data presented for comparison represent the original normalized average Ppal read means per treated genotype.

Statistical analysis of expression level count data with three replicates per treatment was performed using the DEseq package2 (Anders, 2010). Genes were grouped and analyzed in two ways: (1) up or downregulated for each control versus treated combination samples between the three genotypes, (2) constitutive differences in expression between the control sample comparing CCN51/Pound7, ICS1/CCN51, and ICS1/Pound7. For analyses of DEGs, the threshold of *p* ≤ 0.01 was selected to identify genes significantly induced by Ppal treatment and to identify constitutively differentially expressed genes.

The KEGG Automatic Annotation Server was used to obtain KEGG Orthology and KEGG pathways, involving genes differentially expressed between the control and treated samples (Moriya et al., 2007). Gene Ontology (GO) analysis was carried out using the program BLAST2GO (Conesa et al., 2005). Genes were grouped manually based on related functions or pathways, and heat maps of their relative expression were created using CIMminer.^2^ For functional domain analysis, hmmscan^3^ was used to search protein sequences against the Pfam 34.0 database (Mistry et al., 2021).

Genes-encoding carbohydrate-active enzymes were identified using the web server-based automated CAZyme annotation server dbCAN2 (Zhang H. et al., 2018). To establish the similarity between the two publicly available cacao genomes, the Forastero cacao genome v2.1 (Motamayor et al., 2013) was subjected to bidirectional BLAST using the BLASTn search against the Criollo cacao genome v2.0 (Argout et al., 2017). Additionally, the Forastero cacao genome v2.1 was also subjected to bidirectional BLAST using the BLASTn search against the *Arabidopsis thaliana* reference genome (Cheng et al., 2017).

### Transcript Profile Analysis by Machine Learning

Normalized RNA-Seq count data were Log_2_ transformed, and neural network self-organizing map (SOM) analysis (Kohonen, 1990) was employed (using MATLAB Neural Network Toolbox™) to objectively provide an unsupervised multivariate and non-linear classification of the transcriptome into 48 different classes. This process groups entries (genes) based on their patterns across the samples independent of treatments. Three different clustering evaluation criteria were used to identify the optimum number of SOM classes: Calinski–Harabasz index values, Davies–Bouldin index values, and Silhouette values (Ünlü and Xanthopoulos, 2019). This insight was combined with a visual inspection of the classes produced, the fact that our SOM structure was two dimensional, in determining that 48 classes (a 6-x-8 SOM structure) provide the best balance of detail while not over-fitting.

## Results

### Genotype Levels of Resistance in Stage 2 Leaves

**FIGURE 1:**
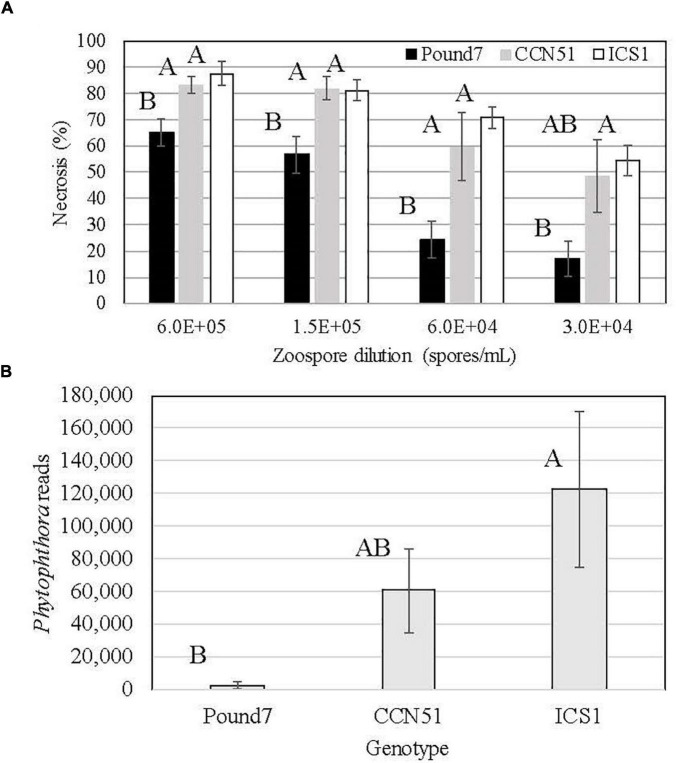
Responses of cacao clones ICS1, CCN51, and Pound7 to *Phytophthora palmivora* (Ppal) infection. **(A)** Ppal-induced leaf necrosis in young red leaves of three genotypes 48 h after inoculation with 20-μl drops of concentration of four zoospores (6.0 × 10^5^, 1.5 × 10^5^, 0.6 × 10^5^, 0.3 × 10^5^ zoospores/ml). Data were analyzed separately for each zoospore dilution, and letters above standard error bars indicate differences (*p* ≤ 0.05). **(B)** Average normalized Ppal reads detected in RNA-Seq libraries for each genotype (ICS1, CCN51, and Pound7) in young red cacao leaves 24 h after inoculation with 20-μl-drop zoospores (0.6 × 10^5^ zoospores/ml). Different letters above standard error bars indicate significant differences (*p* ≤ 0.05).

The three genotypes studied varied in their reactions to Ppal infection (Figure 1A). Pound7 had less necrosis 48 h after infection than ICS1 and CCN51 at all but the 0.3 × 10^5^ zoospore/ml concentration, where Pound 7 was still different from ICS1. The 0.6 × 10^5^ zoospore concentration was chosen for transcriptome analysis, having maximized the differences in infection reactions. We analyzed the18 sequenced transcriptomes for Ppal transcripts. There were differences between the Ppal transcript loads carried by the genotypes (Figure 1B). Pound7, having the lowest Ppal transcript load, was separated from ICS1, which had the highest Ppal transcript load.

### Cacao Transcriptome-General

The primary Forastero cacao transcriptome^4^ consists of 27,379 genes (Table 1). The 18 RNA-Seq libraries averaged 14,720,559 raw reads, with 24,672 transcripts having at least one read among the 18 libraries (Supplementary Data Sheet 1 and Table 1). The cacao transcriptome was compared to the Criollo cacao genome V.2,^5^ taking the 26,001 putative IDs therein. Lastly, we compared the cacao transcriptome to the *Arabidopsis* transcriptome (TAIR^6^ and GenBank accessions CP002684–CP002688), taking 21,543 putative IDs below e ≤ 1 × 10^–10^.

**TABLE 1:** Summary analysis of cacao transcriptome and responses of clones ICS1, CCN51, and Pound7 to *Phytophthora palmivora* 24 h post inoculation.^1^

								Clone					
	Overall cacao transcriptome							ICS1		CCN51		Pound7	
	Total genes	Exp	Exp ≥ 5	Ind	Rep	Ind-All	Rep-All	Ind	Rep	Ind	Rep	Ind	Rep
Total	27,379	24,672	18,722	2,521	1,917	115	6	1,324	437	2,003	1,379	175	253
KEGG pathways	8,257	8,045	7,242	842	810	38	1	386	146	700	628	52	92
Plant pathogen interactions (map04626)	199	193	165	45	6	4	0	32	0	30	6	8	0
PAMP	89	86	74	26	3	4	0	21	0	19	3	4	0
Bacterial flagellin	6	6	6	2	0	0	0	2	0	0	0	0	0
Bacterial Ef-Tu	3	3	3	1	0	0	0	1	0	0	0	0	0
Effector-triggered immunity	78	75	62	15	2	0	0	8	0	8	2	4	0
Fungal effectors	3	3	3	0	0	0	0	0	0	0	0	0	0
Plant hormone signal transduction (map04075)	252	239	190	37	30	2	0	18	16	29	15	3	2
Tryptophan metabolism	110	101	67	19	12	0	0	6	12	15	0	1	0
Zeatin biosynthesis	26	23	17	3	2	0	0	2	0	2	2	0	0
Diterpinoid biosynthesis	9	9	9	0	0	0	0	0	0	0	1	0	0
Carotenoid biosynthesis	35	35	31	2	8	0	0	1	1	2	7	0	2
Cysteine/methionine metabolism	18	18	17	2	2	0	0	2	1	2	1	0	0
Brassinosteroid biosynthesis	24	24	22	3	2	0	0	1	0	2	2	0	0
α-Linolenic acid metabolism	13	13	13	4	2	0	0	4	2	2	0	0	0
Phenylalanine metabolism	17	16	14	4	2	2	0	2	0	4	2	2	0
MAP signaling pathway-plant (map04016)	133	131	115	26	11	5	0	19	2	20	9	5	2
Pathogen infection	35	33	28	15	0	4	0	11	0	11	0	4	0
Pathogen attack	26	25	23	8	0	2	0	4	0	7	0	2	0
Ethylene	34	34	29	5	2	0	0	4	1	4	1	0	0
Jasmonic acid	5	5	5	1	0	0	0	1	0	0	0	0	0
Cold/salt	5	5	5	2	0	0	0	1	0	1	0	0	0
Salt/drought/osmotic stress	34	34	31	2	4	0	0	1	0	2	3	0	2
Ozone	3	3	3	0	0	0	0	0	0	0	0	0	0
Wounding	18	18	15	3	1	1	0	2	0	2	1	1	0
Phenylpropanoid biosynthesis (map00940)	174	164	129	60	11	6	0	40	3	53	5	8	5
Ethylene biosynthesis (M00368)	12	12	12	6	2	1	0	6	1	6	1	1	0
α-Linolenic acid metabolism (map00592)	59	55	47	10	5	1	0	5	0	9	4	1	1
Terpenoid backbone biosynthesis (map00900)	51	50	43	7	1	0	0	7		3	1	0	0
CAZy identities	1,205	1,129	906	219	80	13	0	114	24	182	53	18	10
AA	188	178	130	54	11	8	0	29	4	43	3	14	4
AA1-laccase	41	38	24	11	3	1	0	9	2	4	0	1	1
AA2-peroxidase	80	75	55	23	8	1	0	11	2	21	2	3	3
AA7-FAD-binding Berberine family	39	37	32	15	1	6	0	13	0	13	1	6	0
GH	368	336	265	64	22	2	0	32	9	55	13	3	3
GH18-chitinase	28	24	16	7	0	1	0	6	0	4	0	1	0
GH19-chitinase	11	11	10	7	0	0	0	5	0	7	0	0	0
GT	453	439	379	65	37	2	0	35	7	52	31	2	1
GT1-UDP-glucuronosyltransferase	141	133	109	21	11	2	0	16	3	17	8	2	0
GT31-galactosyltransferase family	18	18	17	5	0	0	0	4	0	5	0	0	0
CE	115	97	69	21	5	0	0	6	2	20	3	1	1
CBM	59	58	49	9	3	1	0	5	1	8	2	2	0
PL	22	20	14	6	2	0	0	1	1	6	1	0	0
SignalP-noTM	139	113	77	19	6	1	0	11	3	15	2	2	1
SignalP-TM	2,231	2,011	1,566	361	105	27	0	204	36	299	62	37	14
Uncharacterized	4,922	3,688	1,277	175	126	8	2	93	37	151	78	12	25
Named-not CAZY, SP, or KEGG	12,342	11,253	8,846	1,185	887	40	3	654	215	895	621	76	125
Transcription factors	1,159	1,093	854	141	101	11	0	84	27	111	68	14	13
WRKY-related	59	59	55	24	3	4	0	21	0	19	3	5	0
ERF-related	111	103	67	19	6	0	0	8	1	18	3	1	2
Calmodulin	73	71	65	17	2	2	0	17	1	8	1	2	0
EF-hand/calcium-binding	99	98	80	9	3	1	0	9	1	5	2	1	0
Myb domain	204	183	132	25	14	1	0	6	4	23	10	1	2
MAPK	52	49	37	6	3	0	0	5	0	2	3	0	0
Glutathione S-transferase	99	90	66	21	3	3	0	14	2	18	1	3	0
Ubiquitin associated	730	697	616	58	77	2	0	36	9	41	63	3	11
Germin-like	45	35	23	18	0	7	0	14	0	15	0	9	0
Pr-10	39	35	19	11	0	4	0	10	0	11	0	4	0
Proteases	338	321	269	49	21	6	0	26	1	38	19	9	3
Lectin-related	305	293	208	82	9	8	0	65	0	49	6	7	4
G-type	162	158	115	52	3	5	0	39	0	38	2	5	3
L-type	48	45	27	12	2	2	0	11	0	10	1	2	1
LysM-type	25	23	21	4	1	1	0	4	0	2	0	1	0
Malectin-type	64	62	44	14	3	0	0	8	0	10	3	2	9
Cysteine-rich RK	97	86	57	21	0	3	0	19	0	14	0	3	0

*Genes were grouped and analyzed by combinations of KEGG and CAZy identity, presence of signal peptides, and putative functions.*

*^1^Exp indicates a number of genes with at least 1 read. Exp ≥ 5 indicates the number of genes with a base mean of at least 5 reads. Ind and Rep indicate the number of genes induced or repressed by Ppal inoculation in at least one clone (p ≤ 0.01), respectively. Ind-All and Rep-All indicate the number of genes induced or repressed by Ppal inoculation in all three clones, respectively.*

There were 2,521 transcripts, showing induction in at least one genotype after *Phytophthora* treatment and 1,917 transcripts showing repression (Table 1). Only six genes were consistently repressed by Ppal infection, while 68 genes were repressed in ICS1/CCN51. There were 115 genes induced in the Ppal-treated samples compared to respective controls for all three genotypes. The Ppal-induced genes varied from 175 (Pound7) to 2,003 (CCN51), while the number of repressed genes varied between 253 (Pound7) and 1,379 (CCN51). Only six genes were downregulated in all three genotypes by Ppal infection. Repressed genes will not be further discussed.

### KEGG Pathways

There were 8,257 genes with KEGG identities spread over 269 pathways (Supplementary Data Sheet 2) with 236 pathways having Ppal-induced genes (Supplementary Data Sheet 1). Thirty-eight genes with KEGG identities were induced by Ppal infection in all three genotypes.

### KEGG Pathways-Signal Perception

#### MAP Signaling Pathway-Plant (map04016)

**FIGURE 2:**
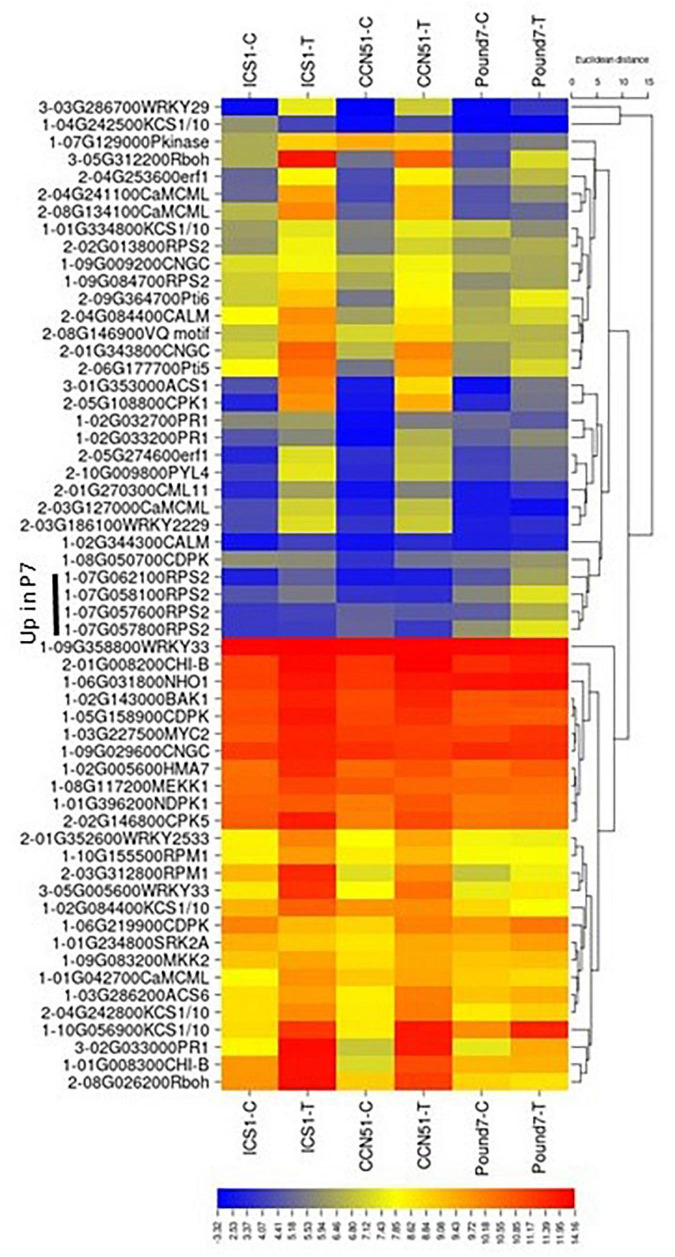
A heat map displaying relative expression levels of induced cacao genes involved in the map-signaling pathway (map04016) and PAMP-mediated and effector-triggered immunity components of the KEGG plant pathogen interactions (map04626) pathway in young red leaves of ICS1, CCN51, and Pound7 24 h after inoculation with 20-μl drops of zoospores (0.6 × 10^5^ zoospores/ml). Initial numbers indicate the number of genotypes with a significantly induced expression between treated and control samples. Numbers represent gene identifications in the cacao transcriptome (Supplementary Data Sheet 1). Terminal letters indicate a gene function/pathway step.

There were 133 transcripts with KEGG IDs associated with the MAP signaling pathway-plant (MSP) map04016 (Table 1). There were 26 MSP transcripts induced by Ppal treatment in at least one genotype and five genes induced by Ppal infection for all three genotypes (Figure 2). Fifteen MSP genes were induced by Ppal infection in ICS1/CCN51. The MSP Pathogen Infection component had 15 genes upregulated, including 4 genes induced in all three genotypes (WRKY TF 33, WRKY TF 29, PR protein 1, and 1-aminocyclopropane-1-carboxylate synthase), followed by the Pathogen Attack component with eight genes upregulated. The only other MSP-associated gene showing induction in all three genotypes putatively encodes a respiratory burst oxidase (Rboh) enzyme. There is a significant overlap of gene functions/identities between the components of the plant MAP signaling pathway in KEGG. The terminal gene in ethylene action putatively encoding an ethylene-responsive TF 1, along with a basic endochitinase B, was induced in ICS1/CCN51 as part of the response to ethylene.

#### Plant Pathogen Interactions (map04626)

There were 199 transcripts with plant pathogen interactions (PPI) map04626 identities (Table 1). There were 45 PPI transcripts induced by Ppal treatment in at least one genotype (Table 1) but only 4 PPI transcripts, all part of the fungal PAMP pathway (Figure 2), induced by Ppal infection for all three genotypes. Additional 21 PPI transcripts were induced by Ppal infection in ICS1/CCN51. Fifteen of these 21 transcripts were associated with the PAMP pathways. Candidates for two steps in the PAMP-associated pathway were missing from the cacao transcriptome analysis (CF9-disease resistance protein and FRK1-senescence-induced receptor-like serine/threonine-protein kinase) (Supplementary Figure 1 in Presentation 1). Four of the steps in the PAMP-associated pathway have candidate genes induced in all three genotypes (Rboh-respiratory burst oxidase, WRKY33, WRKY29, and PR1-pathogenesis-related protein 1), while three additional steps had genes induced in ICS1/CCN51 (Supplementary Figure 1 in Presentation 1).

There were 78 genes associated with the ETI process (Table 1). Fifteen of the ETI genes were induced by Ppal in at least one genotype (Figure 2), seven of which carry NB-ARC motifs. Genes putatively encoding PR genes transcriptional activators Pti5 and Pti6, disease-resistance protein RPM1, disease-resistance protein RPS2, 3-ketoacyl-CoA synthase KCS1/10 were induced by Ppal in ICS1/CCN51.

There are several gene blocks carrying multiple copies of specific PPI genes (Supplementary Data Sheet 1). There is a six-member block of PR protein 1-encoding genes on Chromosome 2, 3 of which are inducible by Ppal. A five-member block of 3-ketoacyl-CoA synthase-encoding genes occurs on Chromosome 4, one of which is inducible by Ppal. A block of the 12 genes-encoding NB-ARC domain containing proteins are found on Chromosome 7, members which are constitutively expressed at higher levels and/or are inducible in Pound7 only (Figure 2).

#### Plant Hormone Signal Transduction (map04075)

**FIGURE 3:**
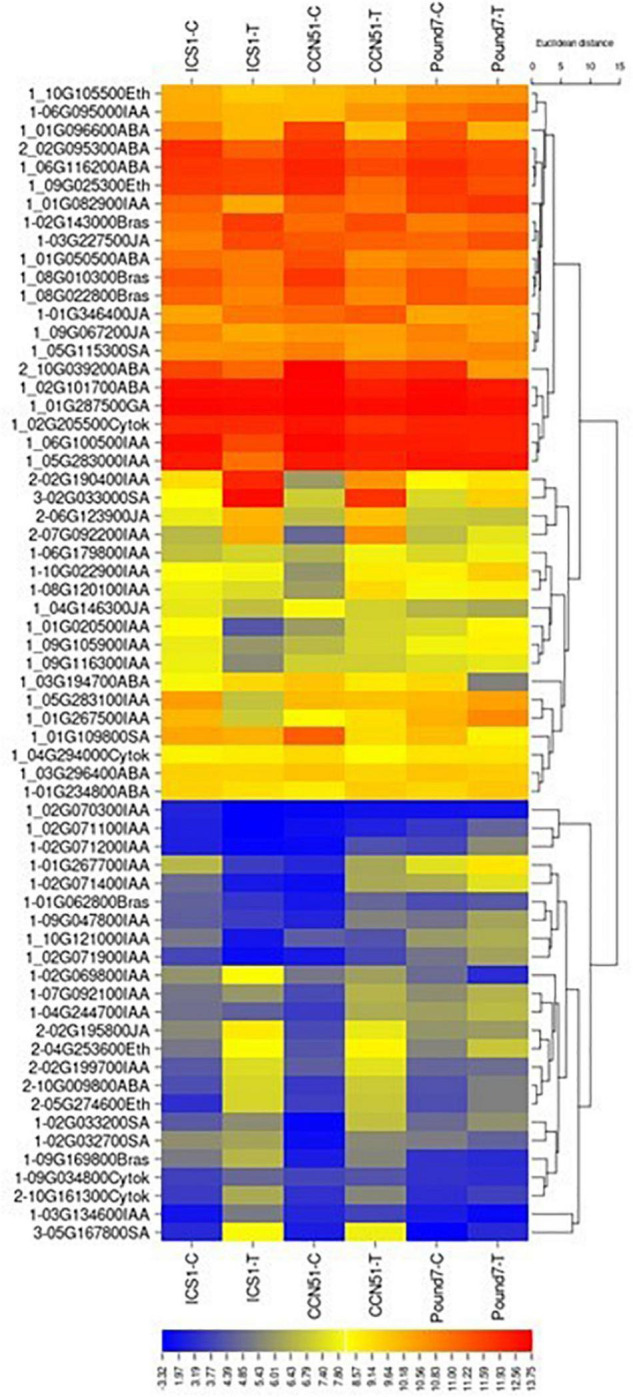
A heat map displaying relative expression levels of cacao genes involved in the Plant Hormone Signal Transduction Pathway (map04075) in young red leaves of ICS1, CCN51, and Pound7 24 h after inoculation with 20-μl drops of zoospores (0.6 × 10^5^ zoospores/ml). Initial numbers indicate the number of genotypes with significant differential expression between treated and control samples. The following – and _, indicate upregulated, downregulated genes, respectively. The following numbers represent gene identifications in the cacao transcriptome (Supplementary Data Sheet 1). Terminal letters indicate hormone/pathway.

There are 252 transcripts with KEGG IDs as part of plant hormone signal transduction (PHST) map04075 (Table 1). There are 37 PHST transcripts induced by Ppal infection in at least one genotype and eleven PHST transcripts induced by Ppal infection in ICS1/CCN51. Two genes were induced by Ppal infection in all three genotypes (Figure 3). Tryptophan metabolism (IAA driven) had the most differentially expressed genes with 19 upregulated, only one of which is in Pound7, and 12 downregulated, none of which were in Pound7, followed by carotenoid biosynthesis (abscisic acid driven) with 2 genes upregulated and 8 downregulated. An ethylene-responsive transcription factor 1 (ERF1) was induced in ICS1/CCN51 as part of cysteine/methionine metabolism (ethylene driven). A gene putatively encoding a ZIM domain-containing protein (JAZ), α-linolenic acid metabolism (jasmonic acid driven), was induced in ICS1/CCN51. Phenylalanine metabolism [salicylic acid (SA) driven] had genes for 2 of the 3 steps induced in all three genotypes, including genes putatively encoding TF TGA and PR1 protein.

### Gene Families-Signal Perception

#### Lectin-Related Proteins

**FIGURE 4:**
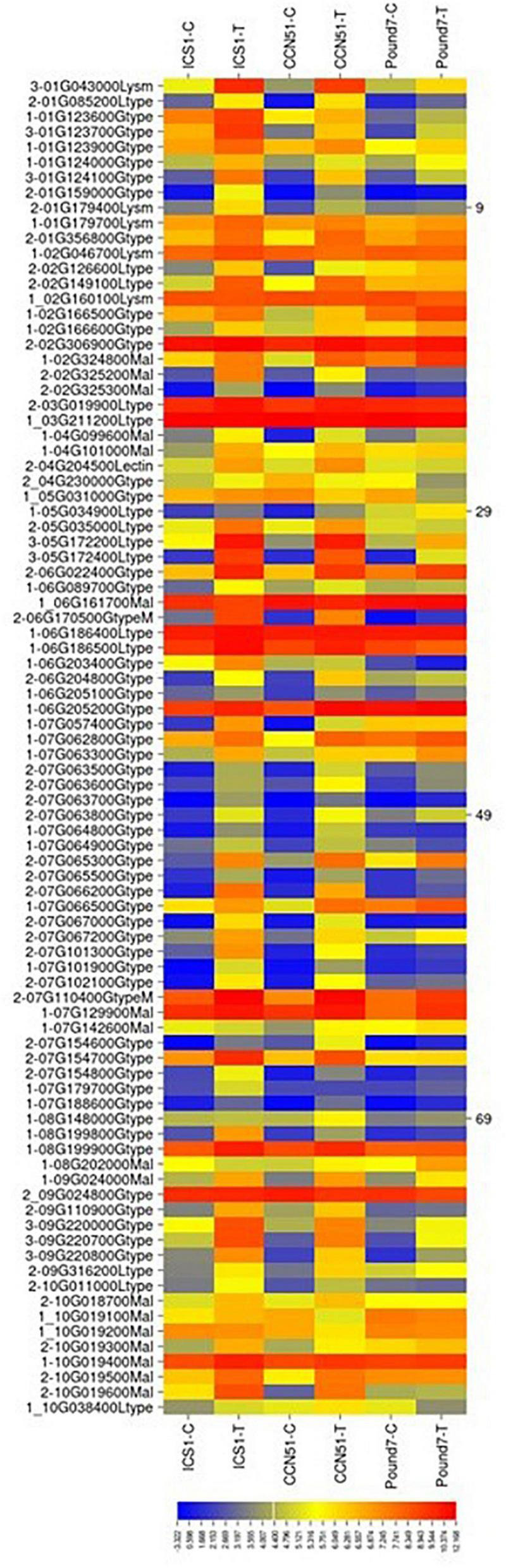
A heat map displaying relative expression levels of cacao gene, putatively encoding lectin-related proteins in young red leaves of ICS1, CCN51, and Pound7 24 h after inoculation with 20-μl drops of zoospores (0.6 × 10^5^ zoospores/ml). Only genes showing differential expression in at least one genotype were included. Initial numbers indicate the number of genotypes with significant differential expression between treated and control samples. The following – and _, indicate upregulated and downregulated genes, respectively. The following numbers represent gene identifications in the cacao transcriptome (Supplementary Data Sheet 1). Terminal letters indicate lectin type: Gtype, G-type; Ltype, L-type; Mal, malectin; LysM, LysM type. GtypeM, mannose binding; Lectin, unclassed lectin.

There were a total of 305 genes (Table 1) putatively encoding lectin-related proteins (principally G-type, L-type, LysM, and malectin carbohydrate-binding domains): 162-G-type, 48-L-type, 25 LysM, and 64 malectin. Eighty-two lectin-related proteins (LRP)-encoding genes, 52-G-type, 12-L-type, 4-LysM, and 14-malectin, were induced in at least one genotype by Ppal infection (Figure 4). Eight LRP-encoding genes were induced in all three genotypes (five encoding G-type lectin S-receptor-like serine/threonine-protein kinases, two encoding L-type lectin-domain containing receptor kinases, and one encoding LysM domain receptor-like kinase 4). Forty-seven LRP-encoding genes were induced in ICS1/CCN51. The induced genes were within large gene blocks with multiple genes often induced within blocks.

#### Cysteine-Rich Receptor Kinase

A total of 97 genes were identified as cysteine-rich receptor kinase (CRK) related (Table 1). Additionally, three CRK-like encoding transcripts (Thecc.01G212600.1, Thecc.06G058600.1, and Thecc.06G111000.1) were induced in the three genotypes (Supplementary Figure 2 in Presentation 1). Twenty-one CRK-encoding genes were induced in ICS1/CCN51.

#### NB-ARC, Leucine-Rich Repeat-Related, and Protein Kinase Genes

A search of the cacao transcriptome based on putative identities identified 498 candidate leucine-rich repeats (LRRs), NB-ARC, Protein kinase candidates (NA-LR-PK) (Supplementary Data Sheet 1). A domain search of these gene sequences identified 89 genes with prominent LRR domains, 170 genes with prominent kinase domains, and 211 genes with prominent NB-ARC domains (Table 2). Genes carrying LRR domains were divided into six classes (1 LRR1, 1 LRR4, 7 LRR6, 50 LLR8, 1 LRR9, and 28 LRRNT_2), and kinases were divided into two classes (110 Pkinase and 60 PkinaseTyr). Up to 20 LRR domain, 20 protein kinase domain, and 21 NB-ARC domain candidate genes were induced by Ppal treatment, depending on the genotype. Many genes were induced only in specific genotypes. There were 24NA-LR-PK genes expressed at constitutively higher levels in Pound7, including 14NB-ARC genes and 7Pkinases. There were 13 NA-LR-PK genes expressed at constitutively higher levels in CCN51, including 9 NB-ARC genes and 4 kinases. There were four NA-LR-PK-related genes expressed at constitutively higher levels in ICS1, including two NB-ARC genes and two LRRs.

**TABLE 2:** Characterization of expression profiles for Pkinase, LRR, and NB-ARC-related genes in the cacao transcriptome in young red cacao leaves 24 h after inoculation with 20-μl-drop zoospores (0.6 × 10^5^ zoospores/ml).^1^

Domain	Total	#Expressed	Response to Ppal infection								
			ICS1		CCN51		Pound7		Unique induced		
			Ind	Rep	Ind	Rep	Ind	Rep	ICS1	CCN51	P7
LRR_1	1	1	0	0	0	0	0	0	0	0	0
LRR_4	1	1	0	0	1	0	0	0	0	1	0
LRR_6	7	7	0	0	1	0	0	0	0	0	0
LRR_8	51	50	8	0	8	0	1	1	5	4	1
LRR_9	1	1	0	0	0	1	0	0	0	0	0
LRRNT_2	28	28	8	0	10	0	0	0	1	3	0
LRR sum	**89**	**88**	**16**	**0**	**20**	**1**	**1**	**1**	**6**	**8**	**1**
NB-ARC	211	207	21	3	14	7	7	2	15	9	7
Pkinase	110	110	15	4	14	0	4	0	11	9	2
Pkinase_Tyr	60	60	5	4	5	3	2	0	2	2	1
Pkinase sum	**170**	**170**	**20**	**8**	**19**	**3**	**6**	**0**	**13**	**11**	**3**
RIX1	1	1	0	0	0	0	0	0	0	0	0
RPW8	1	1	0	0	0	0	0	0	0	0	2
Rx_N	14	12	1	0	1	0	2	0	1	1	0
TIR	12	12	1	1	2	0	0	0	1	2	0
Total	**498**	**491**	**59**	**12**	**56**	**11**	**16**	**3**	**36**	**31**	**13**

**Domain**	**Total**	**#Expressed**	**Constitutive expression**								
			**CCN51/Pound7**		**ICS1/CCN51**		**CCN51/Pound7**		**Constitutive uniques**		
			**Up**	**Down**	**Up**	**Down**	**Up**	**Down**	**ICS1**	**CCN51**	**P7**

LRR_1	1	1	0	0	0	0	0	0	0	0	0
LRR_4	1	1	0	0	0	0	0	0	0	0	0
LRR_6	7	7	1	1	0	0	1	1	0	0	0
LRR_8	51	50	1	4	2	2	4	2	1	0	1
LRR_9	1	1	0	0	0	0	0	0	0	0	0
LRRNT_2	28	28	2	1	2	0	4	0	1	0	0
LRR sum	**89**	**88**	**4**	**6**	**4**	**2**	**9**	**3**	**2**	**0**	**1**
NB-ARC	211	207	27	25	7	22	21	28	2	9	14
Pkinase	110	110	4	11	3	6	4	9	0	2	4
Pkinase_Tyr	60	60	4	8	1	4	2	3	0	2	3
Pkinase sum	**170**	**170**	**8**	**19**	**4**	**10**	**6**	**12**	**0**	**4**	**7**
RIX1	1	1	0	0	0	0	0	0	0	0	0
RPW8	1	1	0	0	0	0	0	0	0	0	0
Rx_N	14	12	2	4	0	3	2	3	0	0	2
TIR	12	12	1	1	1	0	0	0	0	0	0
Total	**498**	**491**	**42**	**55**	**16**	**37**	**38**	**46**	**4**	**13**	**24**

*^1^#Exp indicates a number of genes with at least 1 read. Ind and Rep indicate the number of genes induced or repressed by Ppal inoculation, respectively. Up and down indicate the number of genes with more/less constitutive expression in specific genotype comparisons, respectively. Constitutive uniques indicate the number of genes constitutively expressed at higher levels in the specific genotype indicated.*

### Gene Families-Signal Transduction

#### Transcription Factors

**FIGURE 5:**
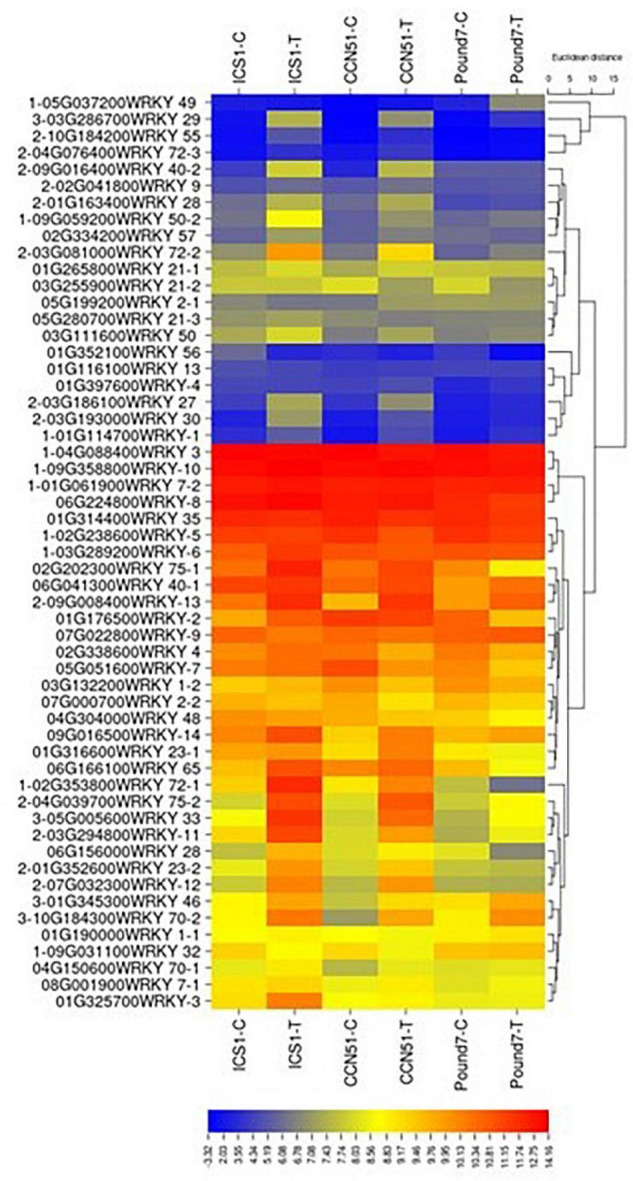
A heat map displaying relative expression levels of cacao gene, putatively encoding WRKY-related proteins in young red leaves of ICS1, CCN51, and Pound7 24 h after inoculation with 20-μl drops of zoospores (0.6 × 10^5^ zoospores/ml). All genes with at least 5 base mean reads are included. Initial numbers indicate the number of genotypes with significant differential expression between treated and control samples and where missing indicates no differential expression. The following – and _, indicate upregulated and downregulated genes, respectively. The following numbers represent gene identifications in the cacao transcriptome (Supplementary Data Sheet 1). The following text identifies putative WRKY identity.

There were 1,159 genes (Table 1), putatively encoding TFs, of which 1,093 were expressed. Of the 1,159 genes, 59 were WRKY TFs (Figure 5), 111 were ERF-related TFs, 73 were calmodulin-related TFs, 99 were EF-hand/calcium-binding-related TFs, and 204 carry a Myb domain, in addition to other diverse gene classes (Table 1). One hundred forty-one putative TF-encoding genes were induced by Ppal in at least one genotype (Figure 5 and Supplementary Figure 3 in Presentation 1). Eleven TF-encoding genes were induced in all three genotypes, with WRKY TFs accounting for 4 of the 11.

All 59 genes (Table 1) encoding WRKY TFs were expressed. Twenty-four WRKY TF-encoding genes were induced by Ppal in at least one genotype (Figure 5). *WRKY46* (Thecc.01G345300.1), *WRKY33* (Thecc.05G005600.1), *WRKY70* (Thecc.10G184300.1), and *WRKY29* (Thecc.03G286700.1) gene were induced in all three genotypes, while 17 *WRKY* genes were induced in ICS1/CCN51. The induced expression levels of *WRKY70* and *WRKY46* were similar in all three genotypes. A *WRKY49* gene was uniquely induced in Pound7 (Thecc.05G037200.1).

### KEGG Pathways-Gene Activation/Induction

#### Ethylene Biosynthesis (M00368)

**FIGURE 6:**
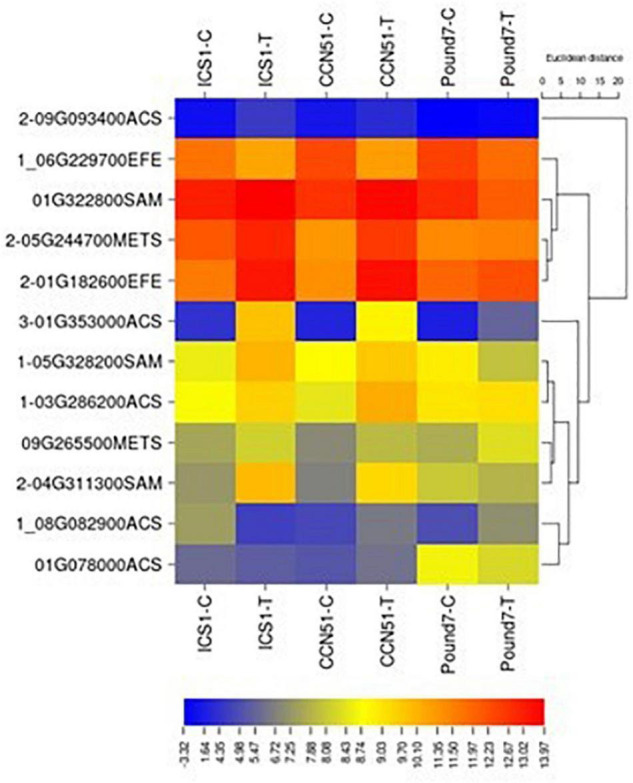
A heat map displaying relative expression levels of cacao genes involved in ethylene biosynthesis (M00017) in young red leaves of genotypes ICS1, CCN51, and Pound7 24 h after inoculation with 20-μl drops of zoospores (0.6 × 10^5^ zoospores/ml). Initial numbers indicate the number of genotypes with significant differential expression between treated and control samples, while genes lacking initial numbers were not differentially expressed. The following – and _ indicate upregulated, downregulated genes, respectively. The following numbers represent gene identifications in the cacao transcriptome (Supplementary Data Sheet 1). Terminal letters indicate the pathway step. ACS, synthase; EFE, aminocyclopropanecarboxylate oxidase; SAM, S-adenosylmethionine synthase; METS-5, methyltetrahydropteroyltriglutamate–homocysteine methyltransferase.

The ethylene biosynthetic pathway is a three-step process within cysteine and methionine metabolism (map00270) and includes S-adenosylmethionine synthetase (SAM1 K00789), 1-aminocyclopropane-1-carboxylate synthase (ACS K20772), and aminocyclopropanecarboxylate oxidase (EFE K05933). Gene members were induced by Ppal for each step in the pathway in ICS1/CCN51 (Figure 6), and one gene, an ACS, was induced by Ppal infection in all three genotypes.

#### α-Linolenic Acid Metabolism (map00592)

There were 59 transcripts associated with α-linolenic acid metabolism (ALAM) map00592 (Table 1). Ten ALAM transcripts were induced by Ppal infection in at least one genotype, and one ALAM transcript was induced in all three genotypes (Supplementary Figure 4 in Presentation 1). Five ALAM transcripts were induced by Ppal infection in ICS1/CCN51.

#### Phenylpropanoid Biosynthesis (map00940)

**FIGURE 7:**
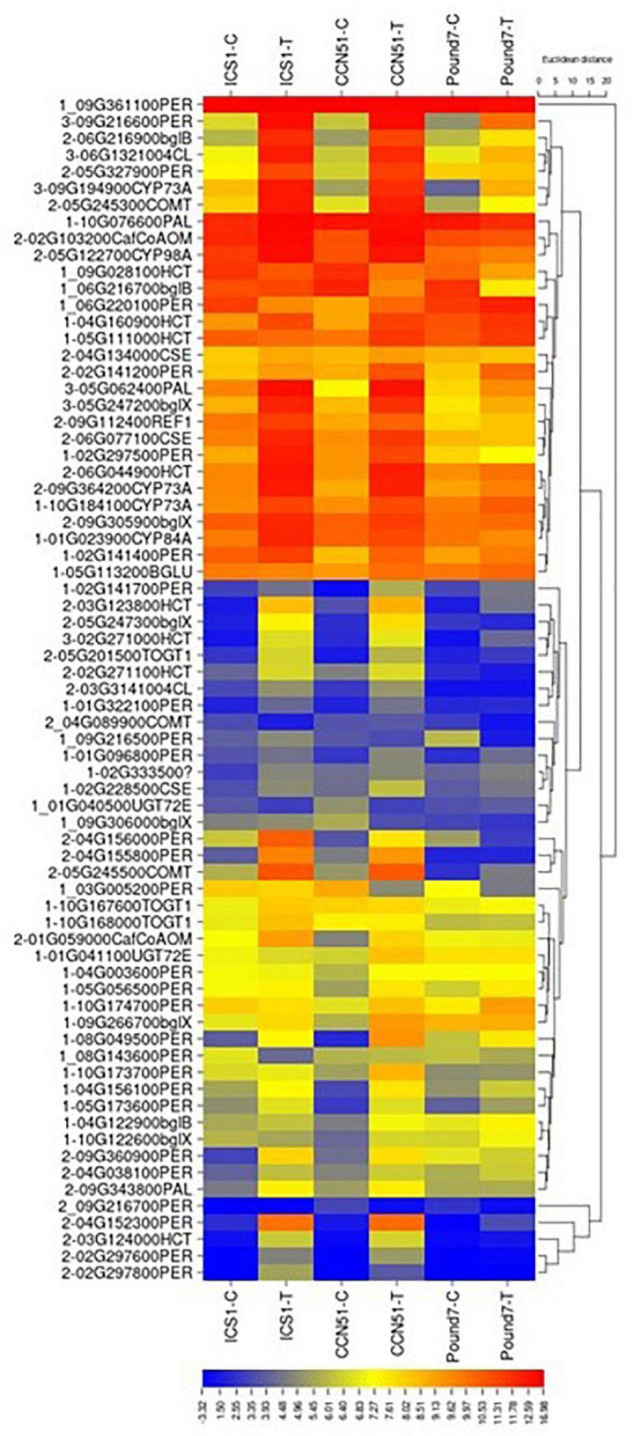
A heat map displaying relative expression levels of cacao genes involved in the Phenylpropanoid Biosynthesis (KEGG map00940) pathway in young red leaves of ICS1, CCN51, and Pound7 24 h after inoculation with 20-μl drops of zoospores (0.6 × 10^5^ zoospores/ml). Initial numbers indicate the number of genotypes with significant differential expression between treated and control samples. The following – and _ indicate upregulated and downregulated genes, respectively. The following numbers represent gene identifications in the cacao transcriptome (Supplementary Data Sheet 1). Terminal letters indicate the gene function/pathway step.

There were 174 transcripts associated with phenylpropanoid biosynthesis (PPB) map00940 (Table 1). There were 60 PPB transcripts induced by Ppal treatment in at least one genotype (Figure 7) and 6 PPB transcripts induced by Ppal infection for all three genotypes. Additional 30 PPBA transcripts were induced by Ppal infection in ICS1/CCN51. There are Ppal-inducible PPB genes for most of the steps in the pathway, beginning with phenylalanine ammonia-lyase and ending with the peroxidases (Supplementary Figure 5 in Presentation 1). Three steps involving cinnamoyl-CoA reductase, cinnamyl-alcohol dehydrogenase, and coniferyl-alcohol glucosyltransferase were not induced. There are blocks carrying multiple copies of specific PPB genes. A block of coniferyl-alcohol glucosyltransferase candidate genes is located on Chromosome 1. Blocks of cationic peroxidase are scattered throughout the genome, many being induced by Ppal infection. There are blocks of candidate shikimate O-hydroxycinnamoyl transferase genes on Chromosomes 5 and 9 that show differences in constitutive expression. A 7-member block of candidate anthocyanidin 3-O-glucosyltransferase-encoding genes are found on Chromosome 1.

#### Terpenoid Backbone Biosynthesis (map00900)

There were 51 transcripts associated with the terpenoid backbone biosynthesis (TBB) map00900 (Table 1). There were seven TBB transcripts induced by Ppal treatment in at least one genotype, and three TBB transcripts were induced by Ppal infection in ICS1/CCN51 (Supplementary Figure 6 in Presentation 1). Two linked putative (+)-delta-cadinene synthase isozyme A genes (Thecc.07G056700.1 and Thecc.07G057000.1) were induced by Ppal infection in all three genotypes, targeting downstream sesquiterpenoid biosynthesis.

### Gene Families-Gene Activation/Induction

#### CAZy Enzymes

There were 1,205 transcripts with CAZy IDs (Table 1). The breakdown of the CAZy groups (Table 1 and Supplementary Data Sheet 1) was 188 auxiliary activities (54 Ppal induced), 368 glucosyl hydrolases (64 Ppal induced), 453 glucosyl transferases (65 Ppal induced), 59 carbohydrate-binding motifs (9 Ppal induced), 115 carbohydrate esterases (21 Ppal induced), 22 polysaccharide lyases (6 Ppal induced). There were 13 CAZy transcripts induced by Ppal treatment in all three genotypes (Supplementary Figure 7 in Presentation 1). Additional 80 CAZy transcripts were induced by Ppal infection in the two most susceptible genotypes. Several CAZy family member groups are highlighted because of their participation in the plant-defense process: AA1 (11 Ppal induced), AA2 (23 Ppal induced), AA7 (15 Ppal induced), GH18 (7 Ppal induced), GH19 (7 Ppal induced), GT1 (21 Ppal induced), and GT31 (5 Ppal induced). There are blocks of AA2-peroxidase on Chromosomes 2, 4, and 9, AA1 laccases on Chromosomes 5 and 9, and AA7FAD-binding Berberine family proteins on Chromosomes 3 and 6.

#### Glutathione-S-Transferases

**FIGURE 8:**
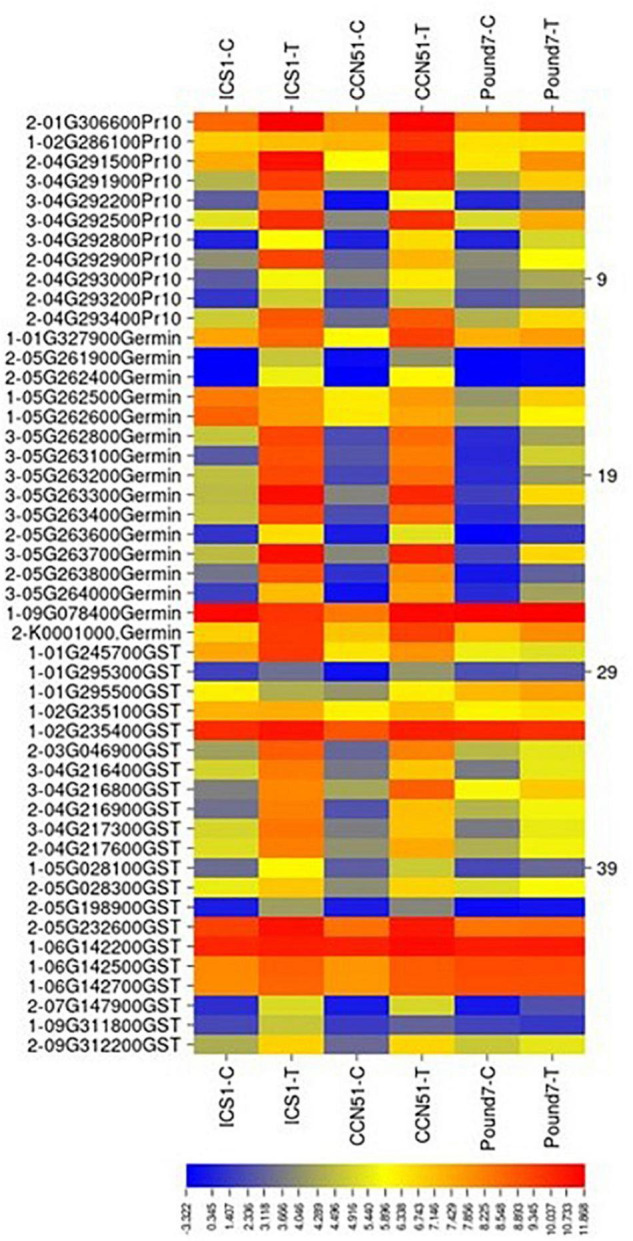
A heat map displaying relative expression levels of induced cacao gene, putatively encoding pathogenesis-related 10 proteins (Pr10), Germin-like proteins (Germin), glutathione S-transferase-related proteins (GST) in young red leaves of ICS1, CCN51, and Pound7 24 h after inoculation with 20-μl drops of zoospores (0.6 × 10^5^ zoospores/ml). Initial numbers indicate the number of genotypes with differential expression between treated and control samples. The following numbers represent gene identifications in the cacao transcriptome (Supplementary Data Sheet 1).

There were a total of 99 genes (Table 1), putatively encoding glutathione-S-transferases (GST) in the current study. Twenty-one *GST*-encoding genes were induced in at least one genotype by Ppal infection (Figure 8). Three *GST*-encoding genes were induced in all three genotypes (all glutathione S-transferase tau 7), all located in a Chromosome 4 gene block consisting of 20 gene members. There are also smaller blocks of 4 or 5 gene members. Eight *GST*-encoding genes were induced in ICS1/CCN51 only.

#### Germin-Like Proteins

A total of 45 transcripts belonged to Germin-like protein (GLP) subfamilies (Table 1). Additionally, seven germin-like-encoding transcripts, all of which are in a single gene block (Thecc.05G263200.1–Thecc.05G264000.1) were induced in the three genotypes (Figure 8). The block includes 22 of 47 GLP -encoding genes identified here.

#### Pathogenesis-Related 10 Proteins

There were a total of 39 genes (Table 1), putatively encoding pathogenesis-related 10 proteins (Pr-10s). Eleven Pr-10-encoding genes were induced in at least one genotype by infection (Figure 8). Four Pr-10-encoding genes were induced in all three genotypes. Six additional Pr-10-encoding genes were induced in ICS1/CCN51. There was a block of Pr-10 genes on Chromosome 4, which included 9 of the 11 Ppal-induced genes.

#### Ubiquitin-Associated Proteins

There were a total of 730 genes (Table 1) encoding ubiquitin-associated proteins (UBA) in the current study. Fifty-eight UBA-encoding genes were induced by Ppal infection (Supplementary Figure 8 in Presentation 1). Only two UBA-encoding genes were induced in all three genotypes (Thecc.01G133700.1-plant U-box 26 and Thecc.09G106400.1-E3 ubiquitin-protein ligase LIN). Twenty UBA-encoding genes were induced in ICS1/CCN51.

#### Proteases/Protease Inhibitors

There were a total of 338 genes (Table 1) putatively encoding Proteases/protease inhibitors (PRO/INH). Forty-nine PRO/INH-encoding genes were induced in at least one genotype by Ppal infection (Supplementary Figure 8 in Presentation 1). Six PRO/INH-encoding genes were induced in all three genotypes (one eukaryotic aspartyl protease, four serine protease inhibitors, and one subtilisin-like protease SBT3.4). Fifteen PRO/INH-encoding genes were induced in ICS1/CCN51. There are blocks of genes-encoding eukaryotic aspartyl protease family proteins on Chromosome 4 and serine protease inhibitors on Chromosome 10.

### Defense-Associated Genes With Genotype-Specific Enhanced Constitutive Differential Expression

There are many differences in constitutive expression levels between the three genotypes studied (Supplementary Data Sheet 1), but the number of genes with higher expression in one genotype compared to the other two genotypes is limited (Table 3): 229 in Pound7, 121 in CCN51, and 182 in ICS1. Although the products of many of these genes could have direct effects on genotype-resistance levels, the genes associated more directly with signal perception and transduction stand out in Pound7. Pound7 has 42 genes putatively encoding lectins, cysteine-rich RKs, LRRs, NB-ARCs, and protein kinases genes with higher constitutive expression compared to 19 genes in CCN51 and 8 genes in ICS1 for these same gene groups. Constitutively differentially expressed genes are spread over all 10 cacao chromosomes for all three genotypes.

**TABLE 3:** Cacao genes are constitutively expressed at higher levels in specific clones (*p* ≤ 0.01).

	Genotype		
Gene grouping	Pound7	CCN51	ICS1
KEGG Id	38	23	49
CAZyme	18	6	9
PAMP	0	0	0
Plant pathogen interactions (map04626)	3	0	4
MAP signaling pathway-plant (map04016)	0	3	0
Plant hormone signal transduction (map04075)	0	2	0
Effector response	3	0	4
Cysteine-rich RK	4	0	0
G- and L-type lectins	5	2	3
LysM lectin	0	0	0
Malectin	3	0	0
LRR/NB-ARC/PK	30	17	5
MAPK	0	0	0
WRKY TF	0	0	0
ERF TF	0	0	0
Calmodulin	0	0	1
Calcium binding/EF-hand	0	0	0
Myb domain	1	0	1
Phenylpropanoid biosynthesis (map00940)	2	1	3
Alpha-Linolenic acid metabolism (map00592)	1	0	0
Terpenoid backbone biosynthesis (map00900)	0	0	0
Pr-10	0	1	0
Germins	1	0	2
Ubiquitin related	6	1	3
Cytochrome p450s	2	5	2
Glutathione S Trans	1	0	1
protease/protease inhibitors	4	6	4
Total genes with constitutive differences	229	121	182

*Genes constitutively expressed at higher levels in specific genotypes were identified and summed into disease-resistance gene groups (Supplementary Data Sheet 1).*

### Self-Organizing Map Analysis

We compared the global cacao gene expression profiles in ICS1, CCN51, and Pound7, responding to Ppal using neural network software to produce SOM classes from the RNA-Seq data. There were 21,266 cacao genes included in the analysis. The transcriptome data were classified into 48 multivariate and non-linear SOM classes (Table 4 and Supplementary Data Sheet 1). The means number of normalized reads for the 48 SOM classes ranged from 1.4 to 22,586. Four classes primarily included genes inducible by Ppal: class 29 (mean reads, 1237.6, 115 genes, 98% inducible), class 43 (mean reads, 32.2, 104 genes, 95% inducible), class 45 (80.0 mean reads, 203 genes, 95% inducible), and class 48 (231.7 mean reads, 165 genes, 93.8% inducible). These same four SOM classes included no downregulated genes. Of the 115 genes induced by Ppal infection in all three genotypes, only 12 genes fell outside these 4 SOM classes. In contrast, the downregulated genes were widely spread among the SOM classes.

**TABLE 4:** Distribution among 48 self-organizing map classes of gene expression profiles for the cacao transcriptome.

	Overall																										
SOM Class	Total genes	Expression (mean reads)	Induced	Repressed	% Induced	% Repressed	Unknowns	Pkinase	LRR	NB-ARC	Malectin RLPK	Lectins	CRK	PAMP mediated	Effector mediated	PrProtein/allergens	WRKY TF	Germins	Ubiquitin related	Glutathione S Trans	Protease	Phenylpropanoid biosynthesis	Plant MAP path	Plant hormones	Linoleic acid metabolism	Terpenoid backbone	Plant path Inter path
1	101	7.9	5	1	5.0	1.0	27							1	1	2			1	1		1		1	2		2
2	246	2.9	3	0	1.2	0.0	61	4		3	3	3			3			1	1		3	4	1	6		1	
3	220	6.9	39	0	17.7	0.0	30	1		1	1	1	3		1	6		1	4	1	4			4			3
4	108	92.5	3	41	2.8	38.0	8	2		2	1	1		1	1	4		1				1	3	5			2
5	228	1,669.8	37	3	16.2	1.3	5	3		1					2			1	3		4	5		3	4		3
6	127	22,586.0	8	25	6.3	19.7	6		1					2	1	1	2		3		3	5	5	1	2		3
7	284	1.4	0	3	0.0	1.1	116			1	1	1			1	2		1	3		2	3	1	2			1
8	264	2.3	0	13	0.0	4.9	100			1	1	1	2		1				5	1		1		5	1		2
9	47	61.8	3	2	6.4	4.3	11	1		7									1				1				1
10	453	498.5	79	18	17.4	4.0	6	6	3							1			9	3	8	4	2	9	2	2	1
11	679	3,078.9	47	148	6.9	21.8	5	2	2					6	4	1	2		36	5	10	4	11	18	4	4	11
12	344	6,725.0	19	66	5.5	19.2	1	1	2	1			2	3	2	2	2		18	1	11	4	3	8	7	2	5
13	96	28.8	10	4	10.4	4.2	21	1	1	3			2		1				2		1	2					1
14	270	1.7	8	0	3.0	0.0	89	1	1		1	1	4	1	2		1		6		2		2	3	1		4
15	187	12.4	4	17	2.1	9.1	22	4		1			1		2	1	1		3		2	2	1	5			2
16	295	228.5	91	11	30.8	3.7	4	4	1	2	2	2	1			2		1	2		7	3		4	1		
17	1,021	670.3	79	133	7.7	13.0	9	8	2	7	3	3	1	1	5	3	3		38	3	20	3	10	10		4	6
18	1,104	1,671.1	86	227	7.8	20.6	15	9	2	4			1	5	4	1	4		69	6	20	5	12	16	4	3	11
19	121	4.3	1	5	0.8	4.1	52	1	2				1		1				1		5	1	1	1			1
20	217	9.3	83	0	38.2	0.0	34	2	2	3	3	3	1			1			6	2	3	2		4			
21	243	27.9	63	3	25.9	1.2	20	4	2	3			1	1	1	6			4		5		1	9		1	2
22	864	120.8	103	25	11.9	2.9	15	5	2	19	1	1	3	2	5	3	2		15	2	8	4	1	4		1	8
23	1,316	1,001.1	97	255	7.4	19.4	12	7	2	2	2	2	2	7		2	3		68	6	21	4	9	8	1	5	7
24	267	1,790.5	16	29	6.0	10.9	10	2			1	1	1	1	1	5	3		6	2	3	7	2	5	3	1	2
25	332	2.1	15	0	4.5	0.0	125	1		2	1	1	2			2	1		2	1	2	2	1	2	2	1	1
26	394	3.7	5	4	1.3	1.0	166	1		1	2	2	2	2	2				5	2	3	4	1	2			4
27	624	14.2	28	28	4.5	4.5	151	6	7	14	2	2	2		4	1			12	1	5	3	2	3		1	7
28	484	77,53	150	2	31.0	0.4	17	17	4	11	4	4	2		2				8	3	10	4	4	8	1		2
29	117	1,237.6	115	0	98.3	0.0	8			1				5	2	5	6		1		6	9	5	4	2		7
30	873	632.2	55	124	6.3	14.2	26	4	6		1	1	1	7	1	4	5		60	2	8	5	4	3	1	3	9
31	330	4.1	10	10	3.0	3.0	124	1		1	2	2	2	1	2	1	2		6		4	4	1	3	1		4
32	522	6.9	20	18	3.8	3.4	175	1	3	2	1	1	3	1	1	2		1	11	2	8	3	1	2			2
33	696	29.0	104	8	14.9	1.1	75	8	5	17			3		5	2		3	8	1	7	4	2	3	1	2	5
34	806	76.6	62	75	7.7	9.3	69	9	3	14	4	4	5	2	2		1		17	3	10	4	4	2		1	5
35	1,043	209.2	109	45	10.5	4.3	22	10	2	10	1	2	1	1	1	3	1		22		16	3	2	7		2	2
36	1,255	419.0	81	172	6.5	13.7	40	5	3	8	1	1	2	1	3		2	1	67	4	13	3	4	6	4	4	5
37	182	3.0	67	0	36.8	0.0	36						1			2	1	5	1	1	2	2					
38	271	13.9	79	0	29.2	0.0	31	5	8	7	1	1	1	1	1	1	1		6		6						2
39	417	29.7	29	40	7.0	9.6	112	6	3	5			2	2	3	2	2		12	1	6	5	4	4		1	7
40	748	44.6	70	36	9.4	4.8	82	6	4	22	4	4	2	3		5	1	1	21	1	9	3		3	2	1	3
41	789	161.5	64	97	8.1	12.3	67	5	1	10	4	4	1	5	2	2	2	1	26	2	14	3	5	6	2	2	8
42	1,191	288.5	72	146	6.0	12.3	42	6	2	6	2	2		4	1		2	1	33	4	13	1	3	8		2	5
43	110	32.2	104	0	94.5	0.0	16		1				1	1		2	1	2	2	1		3	1	1			1
44	218	19.8	14	19	6.4	8.7	48	4	3	1			1	1	1	1	1		7	1		3	1	2	1		2
45	213	80.0	203	0	95.3	0.0	24	2	5	1	2	2	2	5		4	3	5	4	4	3	6	5	4	1		5
46	208	134.3	3	42	1.4	20.2	31		1	4			1	1		1			7			2	2	4			1
47	165	589.4	7	22	4.2	13.3	15	1	1	1			1	3		1	1		1		1	1	4	4	2		4
48	176	231.7	165	0	93.8	0.0	14	1			1	1	3	2	1	3	2	3	10	6	1	5	1	3			3

*Shaded rows indicate SOM classes where genes were predominantly induced by Ppal infection and the shaded columns indicate gene groups associated wih signal perception.*

## Discussion

Pound7 carries resistance to Ppal in leaves in addition to pods (Brown et al., 2007; Gutiérrez et al., 2021). Lesion development and the levels of Ppal transcripts detected in the RNA-Seq libraries were suppressed in Pound7 compared to the susceptible genotype ICS1. Fister et al. (2020) ranked leaves of Pound7 to be the most resistant to Ppal, while ICS1 was ranked the sixth most susceptible to the 60 genotypes compared. Necrosis was reduced up to 65.5% in Pound7 compared to ICS1, while the pathogen transcript levels in ICS1 were 45.9 times higher compared to Pound7 (Figure 1). ICS1 expression of the 115 genes induced in all three genotypes averaged 13.3 times higher than observed in Pound7 but ranged between 1.02 and 53.3 times higher in ICS1, depending on the gene (Supplementary Data Sheet 1). In some cases, expression of induced genes was similar across genotypes despite differences in necrosis and/or pathogen transcript load, whereas, for other genes, induction was proportional to the necrosis level and pathogen transcript load.

Recent work identifying the hydroxycinnamic acid amide clovamide as a major component of resistance in cacao leaves against Ppal infection in cacao genotype SCA6 (Knollenberg et al., 2020) has validated the participation of preformed defense mechanisms in cacao Ppal resistance. ICS1 was also established as a low clovamide-producing genotype. Our Stage 2 leaves approximate the B/C stage in the Guiltinan Laboratory leaf staging system (Fister et al., 2016), which was originally defined as the F-2 stage (Greathouse et al., 1971). The clovamide concentration in both Pound7 and CCN51 is <1% of that detected in healthy (non-infected) Stage C SCA6 leaves (Benjamin Knollenberg, personal communication). Despite these observations, we cannot rule out the potential involvement of other preformed plant defense mechanisms, including other currently uncharacterized metabolites.

### KEGG Pathways Involved in Signal Perception

#### Plant Defense Pathways

The PAMP-mediated component of the PPI pathway (map04626) displayed the most consistent pattern of gene induction among the KEGG signal perception-associated pathways (Figure 2). Candidates for most PAMP-associated components were identified, and several were induced by Ppal infection (Supplementary Figure 1 in Presentation 1). Increases in cytosolic Ca^2+^ are generated by diverse stimuli and stresses through cyclic nucleotide-gated channels (CNGC) (Lecourieux et al., 2006). Among 14 candidate CNGC genes (K05391) identified in the cacao transcriptome, Thecc.01G343800.1 was induced more than 8-times by Ppal infection in ICS1/CCN51. Calcium-dependent protein kinases (CDPKs) and calmodulin-dependent protein kinases (CaMKs) (Cheng et al., 2002) target additional genes that participate in transcriptional reprogramming, the phosphorylation cascade, and synthesis of secondary metabolites (Hashimoto and Kudla, 2011). Two candidates (Thecc.02G146800.1-CPK5 and Thecc.05G108800.1-CPK1) out of 20 CDPKs candidate genes were induced by Ppal infection in genotypes ICS1/CCN51. Two of six calmodulin genes (k02183) were induced by Ppal infection in ICS1/CCN51. Cyclic nucleotide-gated ion Channel 2 (Ali et al., 2007), the WRKY family of TFs (Reddy et al., 2011), and calmodulin-binding protein *60* (Wang et al., 2009) regulate defense responses in *A. thaliana* to pathogen attack. Thecc.02G097700.1, similar to AT2G15760.1 calmodulin-binding protein (DUF1645), was induced by Ppal in all three genotypes (Supplementary Data Sheet 1). CDPKs participate in the phosphorylation and activation of respiratory burst oxidase (Rboh), leading to ROS production (Kobayashi et al., 2007). One of six candidates genes-encoding respiratory burst oxidase (RBOHB K13447) was induced in all three genotypes by Ppal infection. Rboh enzymes act as stress-induced ROS-producing NADPH oxidases, which are essential to systemic ROS signaling (Suzuki et al., 2011). A *WRKY33* candidate (Thecc.05G005600.1) was induced in all three cacao genotypes. In *Arabidopsis*, TF *WRKY33* is essential for defense against necrotrophic fungi (Zheng et al., 2006). Kinetic analyses revealed that loss of *WRKY33* function activated SA-related host responses and elevated SA levels post infection, resulting in higher expression of PR genes, and in the downregulation of jasmonic acid-associated responses (Birkenbihl et al., 2012). A *WRKY 29* (Thecc.03G286700.1-K13426) was also induced by Ppal infection in all three genotypes. *WRKY29* expression was induced by *Fusarium graminearum* (Fg) infection in *Arabidopsis* (Sarowar et al., 2019), and loss of function mutants were more susceptible to infection, while plants constitutively expressing *WRKY29* showed reduced susceptibility. A *Pr-1* gene, in a linked block (Thecc.02G033000.1-K13449), was induced in all three genotypes. PR protein 1 is a marker of plant-defense responses (Van Loon et al., 2006). Fister et al. (2016) also identified a single cacao *Pr-1* on Chromosome 2 that was induced by Ppal and *C*olletotrichum *theobromicola* infection.

There is an overlap between the KEGG plant MAP signaling pathway (map04016, Figure 2) and components of the KEGG PPI pathway (map04626). FLS2 forms a flagellin-induced complex with BAK1, which is essential for the downstream flg-22-signaling responses in *A. thaliana* (Chinchilla et al., 2009). We identified a single BRL-1 receptor kinase (Thecc.02G143000.1-K13416, BAK 1-1) and 13 candidate LRR receptor-like serine/threonine-protein kinase FLS2-encoding genes (K13420) (Supplementary Data Sheet 1). Changes in expression of these genes 24 h after infection are minimal. A candidate MEKK1/BRL1 (Thecc.08G117200.1-K13414), representing the following pathway step, was induced in ICS1. Overall, candidate MAP kinase-encoding genes throughout the cacao genome showed very little response to Ppal infection (Supplementary Data Sheet 1). Only 6 of 52 candidates were induced by Ppal infection and only one (Thecc.01G057700.1-MAPKKK19) of those were induced in two genotypes (ICS1/CCN51). Although the MAP kinase components of these pathways are relatively unchanged, multiple potential downstream components are induced in all three genotypes, including two WRKY TFs (WRKY33 and WRKY29) and end points like PR protein 1, which are also components of the PAMP-mediated pathway.

#### Plant Hormones

Two of five candidate ERF1 TF-encoding genes (Thecc.04G253600.1 and Thecc.05G274600.1), part of the ethylene component of the plant hormone-signaling pathway (KEGG map ko04075), were induced by Ppal infection in ICS1/CCN51, with one copy (Thecc.04G253600.1) just missing the *p* ≤ 0.01 significance cutoff in Pound7 (Figure 3). ERF1 integrates the signals from ethylene and jasmonic acid-guiding induction of defense genes (Lorenzo et al., 2003). A single step, jasmonate ZIM domain-containing protein (JAZ-K13464), in the jasmonic acid component of the mapko04075, also had 2 of 8 candidate genes (Thecc.02G195800.1 and Thecc.06G123900.1) induced in ICS1/Pound7 (Figure 3). In cotton, GhJAZ2 repressed the transcriptional activity of GhMYC2, negatively regulating α-linolenic acid metabolism and JA signaling (Sun et al., 2017). The auxin component of the plant hormone-signaling pathway (Supplementary Data Sheet 2) was altered by Ppal infection in ICS1/CCN51 with 15 candidate genes being induced in CCN51, representing 6 of 7 pathway steps (Figure 3). A gene representing 2 of 3 steps in the SA-component of the plant hormone-signaling pathway was also induced in response to Ppal infection (Figure 3). Expression of the NPR1-related genes, even those with functionally verified activities in cacao plant defense (Shi et al., 2010), was not altered in this study. One of four candidate bZIP (basic leucine zipper)-type TFs (TGA-K14431, Thecc.05G167800.1) was induced by Ppal infection in all three cacao genotypes, as was a Pr-1-encoding gene (Thecc.02G033000.1). Some TGA TFs bind and activate *NPR1* and induce *Pr-1* expression (Zhou et al., 2000).

### Gene Families Associated With Signal Perception

Plant genomes carry large sets of genes-encoding receptor-like kinases (RLKs) and receptor-like proteins (RLPs), which function in signal transduction processes, including functioning as PRRs, detecting molecular patterns that induce plant defense: lectin-associated proteins, cysteine-rich receptor kinases, LRRs, and NB-ABC. We identified large sets of lectins (305), LRRs (89), NB-ARCs (211), protein kinase (170), and cysteine RLKs (52) in the cacao transcriptome (Tables 1, 2). Argout et al. (2011), in publishing the first cacao genome, identified 297 NBS-encoding genes and 253 LRR-RLK-encoding genes. These genes were distributed on all 10 chromosomes and often localized into gene blocks. Mucherino Muñoz et al. (2021) found similar results, identifying genomic regions rich in genes with coiled-coils, nucleotide-binding sites, and LRR domains, the RLK domain, and ginkbilobin2 domains on Chromosomes 1, 3, 4, 6, 8, and 10. Gene polymorphisms within these and other defense-associated genes are expected to contribute to pathogen recognition diversity independent of patterns of gene expression (Pokou et al., 2019).

Lectins are involved in plant cell-to-cell communication. Although their functions are diverse, proteins belonging to the lectin family, especially those with L-type, G-type, LysM, and malectin carbohydrate-binding domains, are known to mediate pathogen perception in plants (Teixeira et al., 2018). Pokou et al. (2019) identified an L-type lectin-encoding gene and a malectin-encoding gene as potentially important in cacao resistance to *Phytophthora megakarya*. Five G-type lectin receptor kinases were induced in all three genotypes by Ppal infection (Supplementary Figure 4 in Presentation 1). The G-type lectin CaMBL1 accumulates in pepper leaves during avirulent *Xanthomonas campestris* pv. *vesicatoria* infection and, when overexpressed in *Arabidopsis*, confers enhanced resistance to *Pseudomonas syringae* pv. *tomato* and *Alternaria brassicicola* (Hwang and Hwang, 2011). In *Arabidopsis*, the G-type lectin CBRLK1 acts as a negative regulator of plant-defense signaling (Kim et al., 2009). Two L-type lectins were induced in all three genotypes. The L-type lectin receptor kinases *LecRK-I.9* or *LecRK-IX.1* in *Arabidopsis* provide resistance to *Phytophthora* and provide *Phytophthora* resistance in transgenic *Nicotiana benthamiana* plants (Wang et al., 2015). The L-like lectin receptor kinase LecRK-I.9 from Arabidopsis is a putative mediator of CW-PM adhesions in *Arabidopsis* and binds to a *Phytophthora infestans* RXLR effector (Bouwmeester et al., 2011). A LysM-type lectin-encoding gene Thecc.01G043000.1, related to *Arabidopsis* AT2G23770-AtLyk4, was induced in all three genotypes. Gong et al. (2020) suggested that AtLYK4 may be a minor chitin receptor or a scaffold stabilizing the chitin receptor complex. Malectin-like receptor kinases can have positive and negative effects on plant immunity, depending on the specific gene involved (Franck et al., 2018).

A total of 87 cacao LRR/NB-ARC/Protein kinase genes were related to *Arabidopsis* genes (Supplementary Data Sheet 1). Although many of these genes showed genotype-dependent differential expression, one protein kinase (Thecc.06G134700.1) was induced in all three genotypes. Perhaps, as interesting, there were 139 of 498 LRR/NB-ARC/protein kinases showing differential constitutive expression. This is relevant since the proteins the genes encode are expected to be prepositioned in the cell (Qi and Innes, 2013). The NB-ARC domain is a conserved nucleotide-binding domain and is thought to act as a molecular switch, cycling between inactive ADP and active ATP-bound forms. The LRR-RKs are composed of an extracellular domain containing the consensus hydrophobic leucine residues, a single membrane-spanning domain, and a cytoplasmic kinase domain with serine/threonine specificity. Ligand binding presumably induces the dimerization or oligomerization of RKs with themselves or co-receptors, activating the intracellular kinase domain and initiating signaling transduction. Some of the LRR-RKs function as heterodimers that form receptor complexes with other LRR-RLPs, which lack the cytoplasmic kinase domain. Both LRR-RKs and LRR-RLPs have been found to play significant roles in plant development and immunity (Qi and Innes, 2013).

Cysteine-rich receptor-like kinases are a large sub-family of plant RLKs. CRKs have a typical RLK domain structure with the addition of DUF26 domains, containing three conserved cysteine residues (Quezada et al., 2019). Three cysteine-rich-related kinases were induced in all three genotypes (Table 1). Two lacked similarities to characterized genes, but one (Thecc.06G058600.1) is related to *Arabidopsis* CRK2. In *Arabidopsis*, CRK2 forms a complex with the NADPH oxidase Respiratory Burst Oxidase Homolog D (RbohD). CRK2 is required for elicitor-induced ROS burst (Kimura et al., 2020). We identified a RbohD that was induced by Ppal infection ICS1/CCN51 and a RbohB that was induced in all three genotypes.

### Gene Families Involved in Signal Transduction

A genome-wide analysis of the *T*. *cacao* WRKY TF family identified 61 WRKY sequences (Silva Monteiro de Almeida et al., 2017). We identified 59 candidate WRKY TFs. Twenty-four WRKY TFs were inducible by Ppal infection, 17 being induced by Ppal infection in ICS1/CCN51, and 4 being induced in all three genotypes (Figure 5): *WRKY46* (Thecc.01G345300.1), *WRKY 33* (Thecc.05G005600.1), *WRKY 70* (Thecc.10G184300.1), *WRKY 29* (Thecc.03G286700.1), and a putative WRKY49 (Thecc.05G037200.1) was induced only in the resistant clone Pound7. *WRKY33* and *WRKY29* are described above. The cacao *WRKY70* (Tc10_p016570) was cited for its potential importance in cacao’s response to *M*oniliophthora *perniciosa* infection (Silva Monteiro de Almeida et al., 2017). *WRKY70* overexpression in *Arabidopsis* enhanced resistance to the biotroph *Erysiphe cichoracearum* and reduced resistance to the necrotroph *A. brassicicola*, serving a role in the balance between SA- and JA-mediated resistance (Li et al., 2006). In *Arabidopsis*, *WRKY46* was selectively induced by SA and the biotrophic pathogen *P. syringae* infection. Increasing combinations of loss of function mutants for *WRKY46*, *WRKY70*, and *WRKY53* displayed progressively greater susceptibility to *P. syringae* infection, leading to the conclusion that the three WRKY genes cooperate in the *Arabidopsis* basal-resistance response (Hu et al., 2012). *WRKY49*-related genes participate in both biotic and abiotic stress tolerance (Wani et al., 2021).

### KEGG Pathways and Gene Induction/Activation

#### Ethylene Biosynthesis

The biosynthesis and the action of plant hormones are often disrupted during infection and resistance responses (Bari and Jones, 2009). Ethylene biosynthesis is an early event during plant pathogen interactions (Boller, 2018) and induces the production of PR proteins (Van Loon et al., 2006). The plant hormone ethylene has a three-step biosynthesis (Polko and Kieber, 2019), including S-adenosylmethionine synthetase, 1-aminocyclopropane-1-carboxylate synthase, and aminocyclopropanecarboxylate oxidase. Members of all three steps were induced by Ppal infection in ICS1/CCN51, and a candidate gene-encoding ACC synthase (Thecc.01G353000.1) was induced in all three genotypes (Figure 6).

#### Phenylpropanoid Biosynthesis

A candidate phenylalanine ammonia-lyase (*PAL*) gene is induced by Ppal infection in all three cacao genotypes. *PAL* catalyzes the first step of phenylpropanoid metabolism (Vogt, 2010). Candidate genes for most steps, in general, phenylpropanoid metabolism, are induced by Ppal infection in all three genotypes (Figure 7 and Supplementary Figure 5 in Presentation 1). Cinnamoyl-CoA reductase, cinnamyl-alcohol dehydrogenase, and coniferyl-alcohol glucosyltransferase were not induced. The phenylpropanoid pathway participates in many processes required for secondary product production. Lignin synthesis is essential for the strengthening of cell walls (O’Malley et al., 1992). Up to 21 candidate genes-encoding peroxidases tied to lignin formation were induced by Ppal infection, mostly in ICS1/CCN51. A single peroxidase (Thecc.09G216600.1) was induced in all three genotypes, although two peroxidases (Thecc.10G174700.1 and Thecc.02G141200.1) were induced in CCN51 and Pound7 and not ICS1. Cell wall peroxidases (Class III peroxidase) participate in the generation of reactive oxygen and nitrogen species (Van Loon et al., 2006), oxidizing hydroxy-cinnamyl alcohols into free radical intermediates and oxidizing phenols (Chittoor et al., 1997). A gene putatively encoding phenylcoumaran benzylic ether reductase (Thecc.06G002300.1-K23050) is induced in all three genotypes with a potential function linked to lignin biosynthesis (Mijnsbrugge et al., 2000). A putative polyphenol oxidase gene (Thecc.06G192400.1) was induced in all genotypes. Polyphenol oxidases function in plant defense by multiple mechanisms (Zhang and Sun, 2021).

### Gene Families and Gene Induction/Activation

Many gene families highlighted here are characterized as PR proteins due to their inducibility and association with responses to infections in diverse plant species (Teixeira et al., 2014; Fister et al., 2016). The peroxidases (Axillary Activities 2, AA2) were described above (Supplementary Figure 7 in Presentation 1). A putative AA1 ascorbate oxidase (Thecc.01G106900.1) was induced in all three genotypes. Ascorbate oxidase is thought to modulate the redox control of the apoplast and influence plant defense through the regulation of signaling cascades (Pignocchi et al., 2006). Three cacao genes, putatively encoding AA7 tetrahydroberberine (THB) oxidase, were induced in all three genotypes by Ppal infection. THB oxidase is the last enzyme of the benzylisoquinoline alkaloids (BIA) pathway, catalyzing berberine (Hagel and Facchini, 2013), BIAs possessing antimicrobial activity (Hagel and Facchini, 2013). Three genes-encoding putative AA7 FAD-binding Berberine family proteins were induced by Ppal infection in all three genotypes. Berberine bridge-like enzyme (BBE) family proteins have diverse activities. Two BBE family members in *Arabidopsis* were identified as monolignol oxidoreductases, playing a role in monolignol metabolism and lignin formation (Daniel et al., 2015). A second *Arabidopsis* study identified 4 BBE-like enzymes as oligogalacturonide oxidases (Benedetti et al., 2018).

Only one GH3 (Thecc.05G247200.1 β-glucosidases) and one GH18 (Thecc.09G331900.1 chitinase 1) were induced in all three genotypes (Supplementary Figure 7 in Presentation 1). In *Arabidopsis*, the GH3-like gene family enzymes adenylate plant hormones, sometimes catalyzing hormone conjugation to amino acids (Zhang C. et al., 2018). Chitinases are induced during pathogen infection, including *Phytophthor*a infection (Cletus et al., 2013; Fister et al., 2016; Ali et al., 2017). Transient expression of chitinase in cacao leaves increased resistance to *Phytophthora tropicalis* (Fister et al., 2016). Two glucosyl transferases (Thecc.09G084000.1 and Thecc.04G094100.1) were induced in all three genotypes, both belonging to the GT1 UDP-glycosyltransferase superfamily (Supplementary Figure 7 in Presentation 1). Enzymes in the UDP-glycosyltransferase superfamily GT1 play roles in plant natural product diversification (Louveau and Osbourn, 2019).

Glutathione S-transferases conjugate glutathione to electrophilic small molecules, forming more soluble peptide derivatives (Estévez and Hernández, 2020). Three genes putatively encoding tau7 class glutathione-S-transferases were induced in all three genotypes by Ppal infection (Figure 8). Tau7 class glutathione-S-transferases are inducible in response to multiple stresses and hormones (Zhou and Goldsbrough, 1993; Sappl et al., 2004). GSTs activity can detoxify substances and attenuate oxidative stress (Estévez and Hernández, 2020).

Germins and GLPs are PR proteins 15 and 16, respectively (Fister et al., 2016). We identified 47 cacao genes putatively encoding Germin/GLP, 7 of which, in a gene block on Chromosome 5, were induced by Ppal infection in all three genotypes (Figure 8). Bernier and Berna (2001) described three functions for Germins and GLP: active enzymes (oxalate oxidase or superoxide dismutase), structural proteins, and receptors. Germin oxalate oxidase activity was implicated in the production of H_2_O_2_ produced by the degradation of calcium oxalate produced by *M. perniciosa* in cacao (Dias et al., 2011).

Four PR protein 10.5’s located on Chromosome 4 were induced in the three genotypes. Fister et al. (2016) identified 23 *Pr-10* genes, eight of which were induced in cacao leaves by Ppal. The same large gene block we observed on Chromosome 4 (Figure 8) was also identified (Fister et al., 2016). Thirteen *Pr-10* members were induced by Ppal infection in pods 3 days after inoculation (Ali et al., 2017).

Two ubiquitin-associated (Table 1) genes were induced in all three genotypes (Supplementary Figure 8 in Presentation 1): aU-box domain-containing protein 26 (*PUB26*-Thecc.01G133700.1) and an E3 ubiquitin-protein ligase LIN/Transducin/WD40-like superfamily protein (Thecc.09G106400.1). Studies have shown that *PUB26s* play prominent roles in plant stress responses by targeting inactive forms of the immune regulatory factor BIK1 for ubiquitination (Wang et al., 2018). Some E3 ubiquitin-protein ligase LIN members are involved in responses to phytohormones and abiotic stresses (Qanmber et al., 2018).

## Conclusion

**FIGURE 9:**
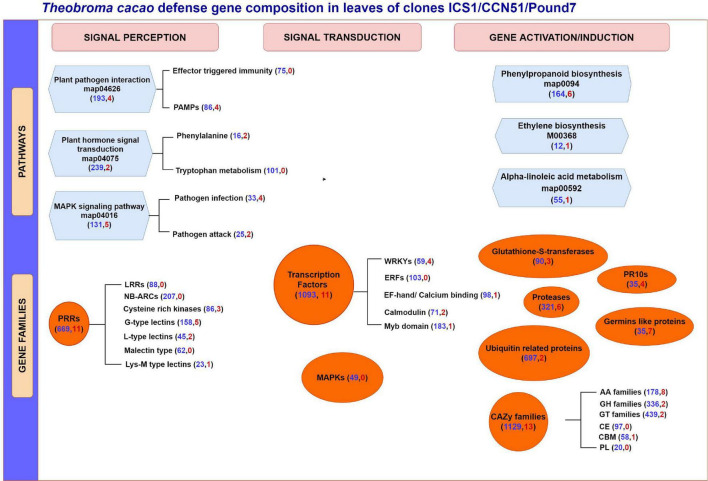
A summary of *T. cacao* defense gene expression in immature leaves of three genotypes (ICS1/CCN51/Pound7) 24 h after infection by *Phytophthora palmivora*. Figure group genes based on their potential participation in specific KEGG gene pathways and different gene families. Genes are further separated based on their perceived participation in signal perception, signal transduction, or gene induction/activation. Numbers in parentheses indicate the total number of genes expressed in the cacao transcriptome (blue) and a total number of genes induced in all three genotypes (red), respectively. PAMPs, pathogen associated molecular patterns; PRR, pathogen recognition receptors; LRR, leucine-rich repeat; NB-ARC, nucleotide-binding adaptor shared by APAF-1,R proteins and CED-4 domain; EF hand, elongation factor hand domain; LysM, lysine M motif; ERF, ethylene responsive factor; MAPK, mitogen-activated protein kinase; PR10s, pathogenesis-related proteins; CAZy, carbohydrate-active enzymes; AA, Auxiliary Activities; GH, Glycoside hydrolase; GT, glycosyl transferases; CE, carbohydrate esterases; CBM, carbohydrate-binding modules; PL, polysaccharide lyases.

More than seven times as many genes were induced in the susceptible genotypes ICS1 and CCN51 than in the resistant genotype Pound7 (Table 1). Over 3,000 genes were induced in cacao pod pieces 24 h after inoculation with Ppal or *P. megakarya* zoospores, many of which fall within the same KEGG pathways and gene families identified here (Ali et al., 2017). Only 115 genes were induced in all three genotypes studied here. We suggest these represent genes that are most sensitive to Ppal infection. This group could be extended by adding genes included among the 616 genes in SOM Classes 29, 43, 45, and 48 (Table 4). These 115 genes include members of gene classes expected to participate in multiple aspects of the cacao defense response from pathogen signal perception and transduction through direct action against the pathogen (Figure 9).

Stage 2 leaves of genotype Pound7 carry resistance to infection by *P. palmivora* (Figure 1). For the most part, the defense-associated genes induced in the resistant genotype Pound7 were also induced in ICS1/CCN51. Yet, the proportional level of induction did vary depending on the gene considered. For the 115 cacao genes induced in all three genotypes, the ratio of mean expression levels in the Ppal-treated samples ranged between 1 and 53 times higher in ICS1 than Pound7, despite ICS1 having more severe necrosis and 46 times greater Ppal transcript load compared to Pound7. Genes do not always respond to infection proportionally, raising the possibility that some genes are responding to Ppal infection more strongly in Pound7. There were also defense-associated genes constitutively differentially expressed at higher levels in specific genotypes, possibly providing a prepositioned defense (Table 3). These genes are spread across all 10 cacao chromosomes and necessarily include chromosomes, carrying the many QTLs identified as contributing to Ppal resistance (Lanaud et al., 2009).

The transcriptome of cacao displays a complex pattern of differential gene expression, which includes genes induced by infection and constitutively expressed genes, many of which have the potential to contribute to disease resistance (Figure 9). Hidden within these expression patterns, gene sequence polymorphisms occur with the potential to alter gene product function/specificity (Pokou et al., 2019). These genes include participants in multiple pathways associated with pathogen signal perception and transduction, resulting in gene activation and, in some cases, enhanced gene expression. Multiple large classes of potential PRRs have been identified, some of which are constitutively differentially expressed among the genotypes tested, and others of which are inducible by Ppal infection. Large classes of genes expected to participate in signal transduction have also been identified. We ended with gene classes-encoding proteins expected to act directly as enzymes in biosynthetic pathways or potentially having direct action against pathogens, many of which are highly inducible in response to pathogen attack. A theme common throughout many of the defense gene classes studied is their occurrence in gene blocks (Legavre et al., 2015; Fister et al., 2016) where individual members are constitutively expressed at different levels, and some members are responsive to Ppal infection. This provides an opportunity for widespread diversity of response to disease processes and provides potential gene sources for the many QTLs identified in cacao as contributing to resistance.

## Data Availability Statement

The original contributions presented in the study are publicly available. This data can be found here: GenBank, BioProject ID: PRJNA785999.

## Author Contributions

BB and SA provided intellectual and editorial comments. BB conceived and designed the experiments. IB performed the experiments. IB, SA, JS, and DL analyzed the data. IB and BB wrote the manuscript. All authors contributed to the article and approved the submitted version.

## Conflict of Interest

The authors declare that the research was conducted in the absence of any commercial or financial relationships that could be construed as a potential conflict of interest.

## Publisher’s Note

All claims expressed in this article are solely those of the authors and do not necessarily represent those of their affiliated organizations, or those of the publisher, the editors and the reviewers. Any product that may be evaluated in this article, or claim that may be made by its manufacturer, is not guaranteed or endorsed by the publisher.

## Footnotes

1
https://usegalaxy.org/

2
http://discover.nci.nih.gov/cimminer

3
https://www.ebi.ac.uk/Tools/hmmer/search/hmmscan

4
https://jgi.doe.gov/improving-cacao-genome-phytozome/

5
https://www.ncbi.nlm.nih.gov/assembly/GCF_000208745.1/

6
https://www.arabidopsis.org/
