# Supplementary material for: Changes in Gene Expression in Leaves of Cacao Genotypes Resistant and Susceptible to Phytophthora palmivora Infection
Source: Front Plant Sci. 2022 Feb 8;12:780805. doi: 10.3389/fpls.2021.780805 (PMC8861199; doi:10.3389/fpls.2021.780805)
Supplement: Supplementary file 3 [file Presentation_1.PPTX]

## Slide 1
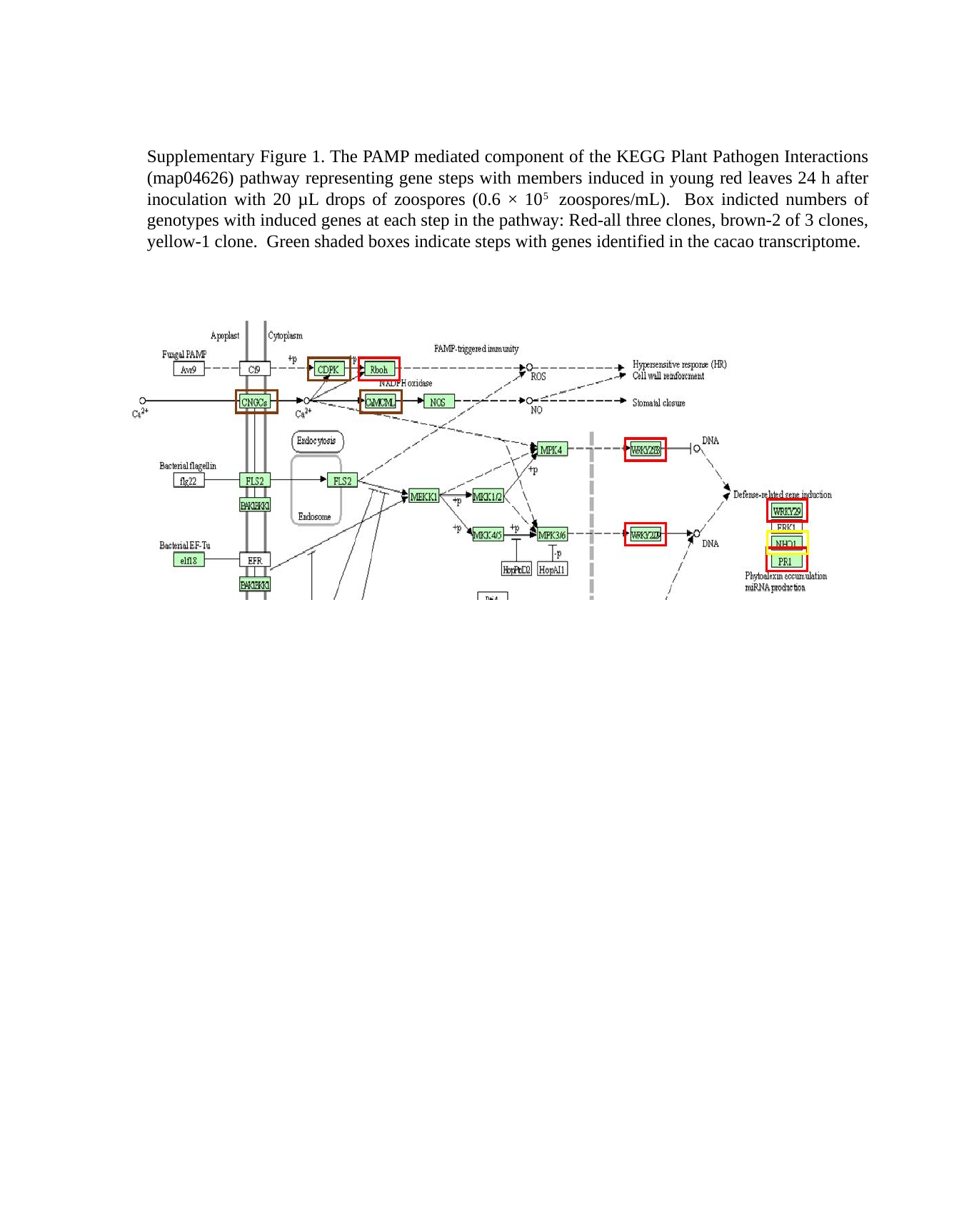

Supplementary Figure 1. The PAMP mediated component of the KEGG Plant Pathogen Interactions (map04626) pathway representing gene steps with members induced in young red leaves 24 h after inoculation with 20 µL drops of zoospores (0.6 × 10^5^ zoospores/mL). Box indicted numbers of genotypes with induced genes at each step in the pathway: Red-all three clones, brown-2 of 3 clones, yellow-1 clone. Green shaded boxes indicate steps with genes identified in the cacao transcriptome.

## Slide 2
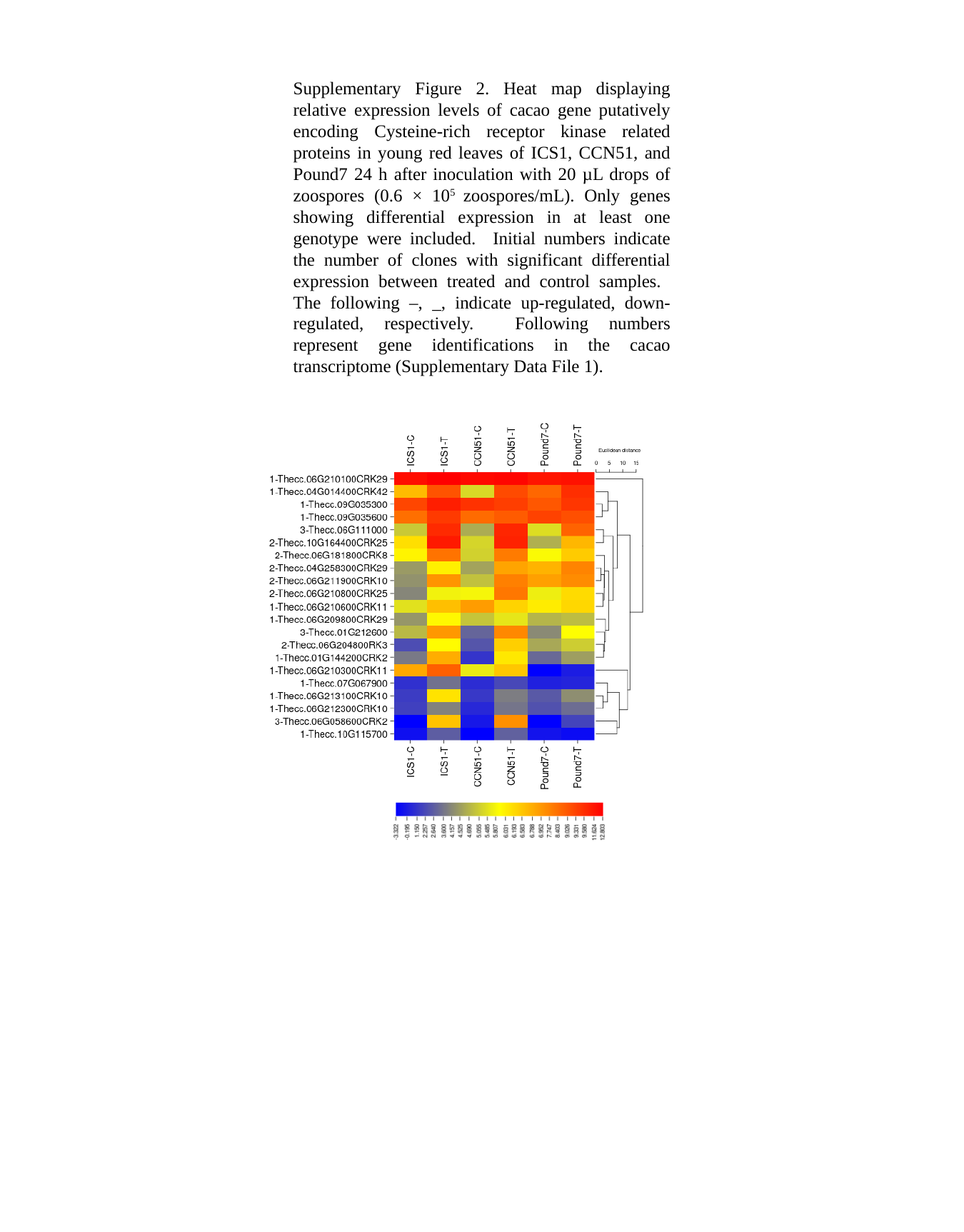

Supplementary Figure 2. Heat map displaying relative expression levels of cacao gene putatively encoding Cysteine-rich receptor kinase related proteins in young red leaves of ICS1, CCN51, and Pound7 24 h after inoculation with 20 µL drops of zoospores (0.6 × 10^5^ zoospores/mL). Only genes showing differential expression in at least one genotype were included. Initial numbers indicate the number of clones with significant differential expression between treated and control samples. The following –, _, indicate up-regulated, down-regulated, respectively. Following numbers represent gene identifications in the cacao transcriptome (Supplementary Data File 1).

## Slide 3
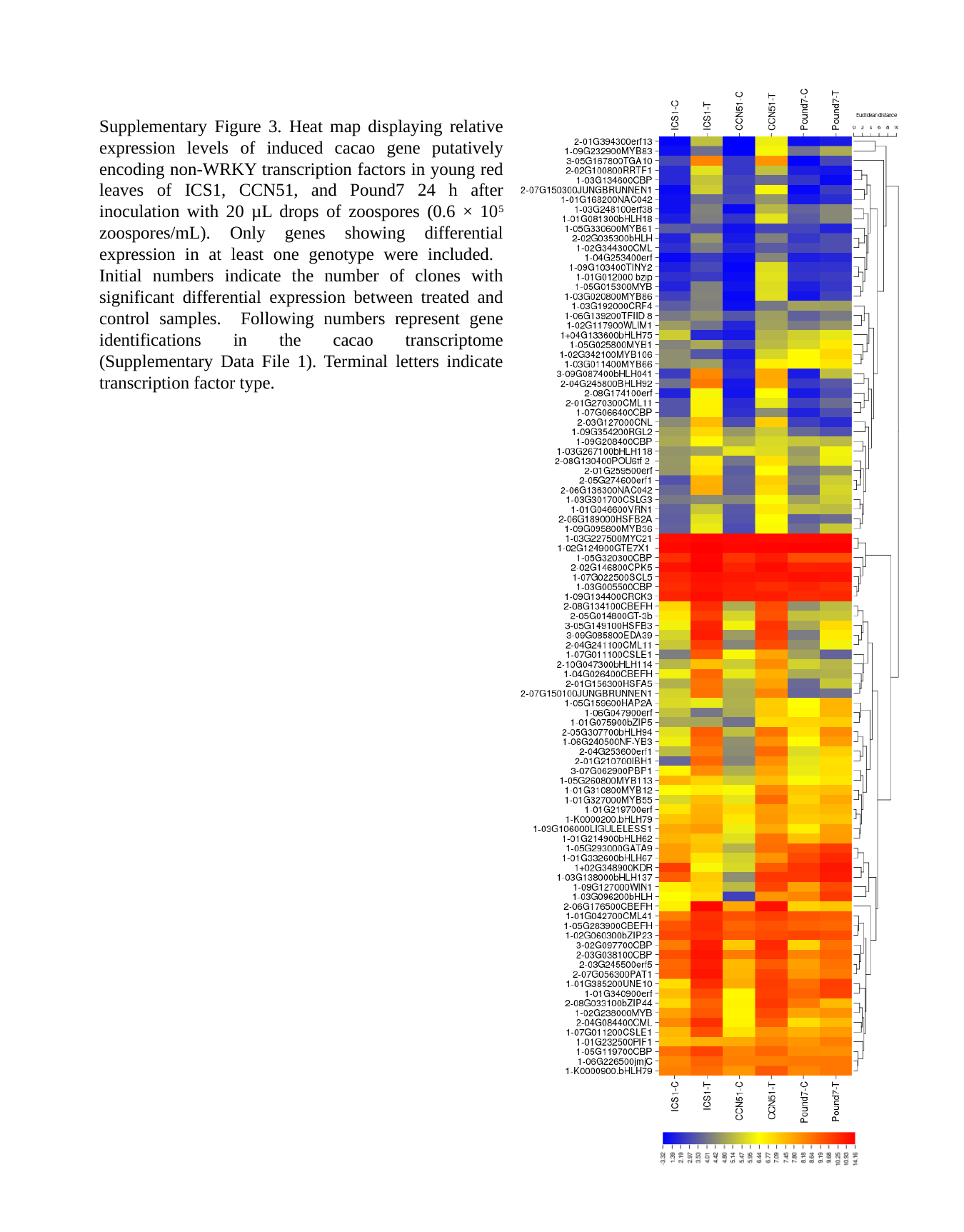

Supplementary Figure 3. Heat map displaying relative expression levels of induced cacao gene putatively encoding non-WRKY transcription factors in young red leaves of ICS1, CCN51, and Pound7 24 h after inoculation with 20 µL drops of zoospores (0.6 × 10^5^ zoospores/mL). Only genes showing differential expression in at least one genotype were included. Initial numbers indicate the number of clones with significant differential expression between treated and control samples. Following numbers represent gene identifications in the cacao transcriptome (Supplementary Data File 1). Terminal letters indicate transcription factor type.

## Slide 4
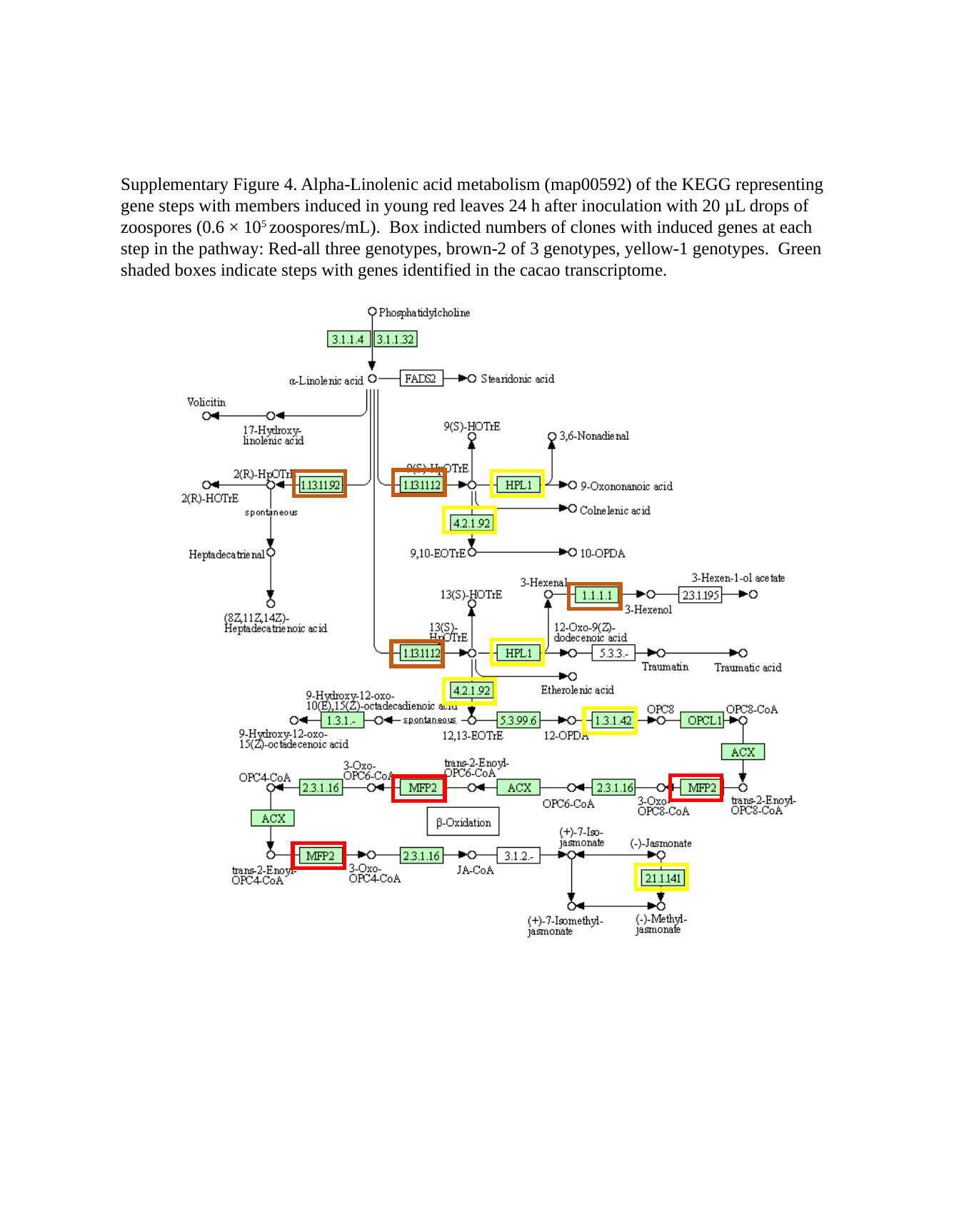

Supplementary Figure 4. Alpha-Linolenic acid metabolism (map00592) of the KEGG representing gene steps with members induced in young red leaves 24 h after inoculation with 20 µL drops of zoospores (0.6 × 10^5^ zoospores/mL). Box indicted numbers of clones with induced genes at each step in the pathway: Red-all three genotypes, brown-2 of 3 genotypes, yellow-1 genotypes. Green shaded boxes indicate steps with genes identified in the cacao transcriptome.

## Slide 5
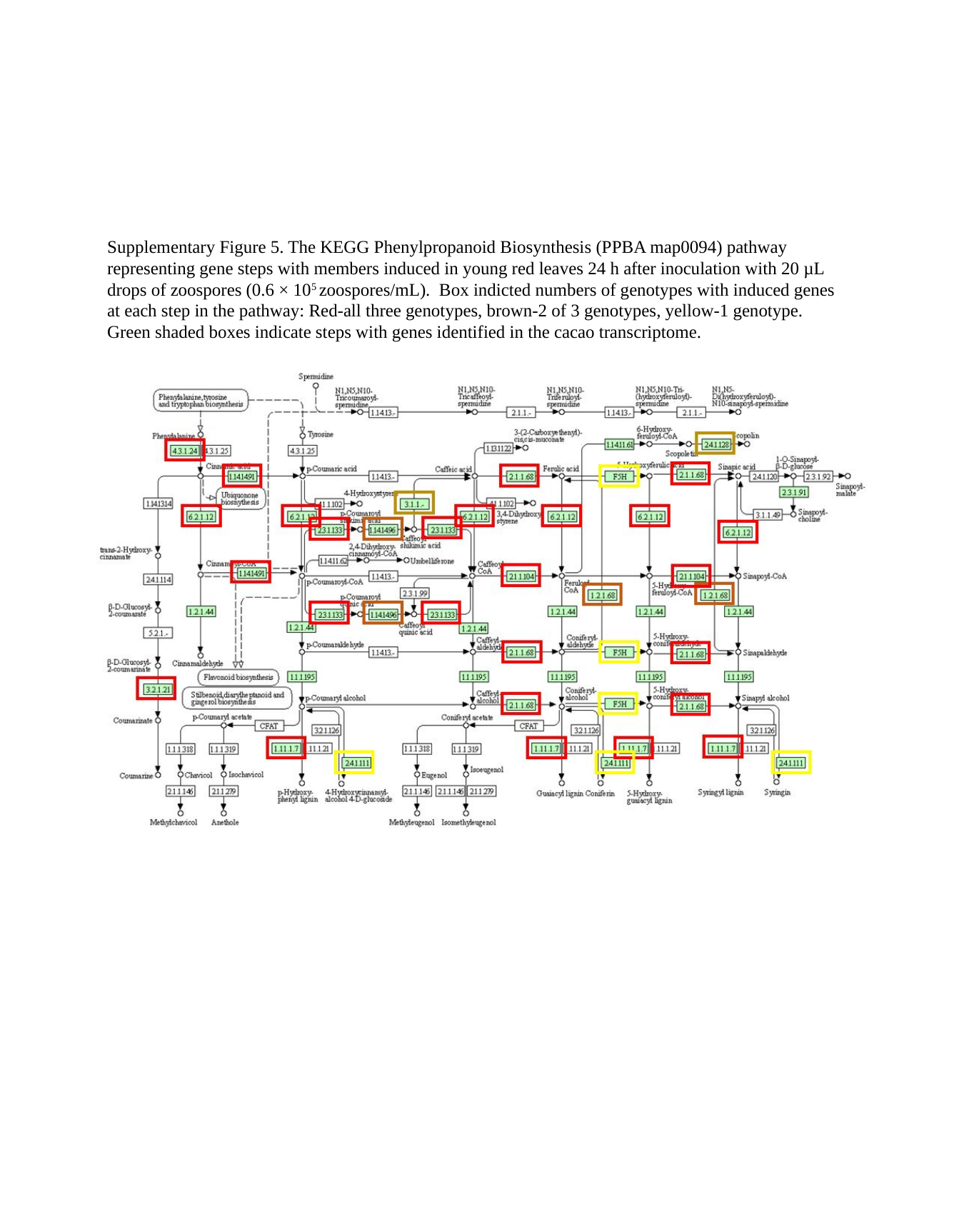

Supplementary Figure 5. The KEGG Phenylpropanoid Biosynthesis (PPBA map0094) pathway representing gene steps with members induced in young red leaves 24 h after inoculation with 20 µL drops of zoospores (0.6 × 10^5^ zoospores/mL). Box indicted numbers of genotypes with induced genes at each step in the pathway: Red-all three genotypes, brown-2 of 3 genotypes, yellow-1 genotype. Green shaded boxes indicate steps with genes identified in the cacao transcriptome.

## Slide 6
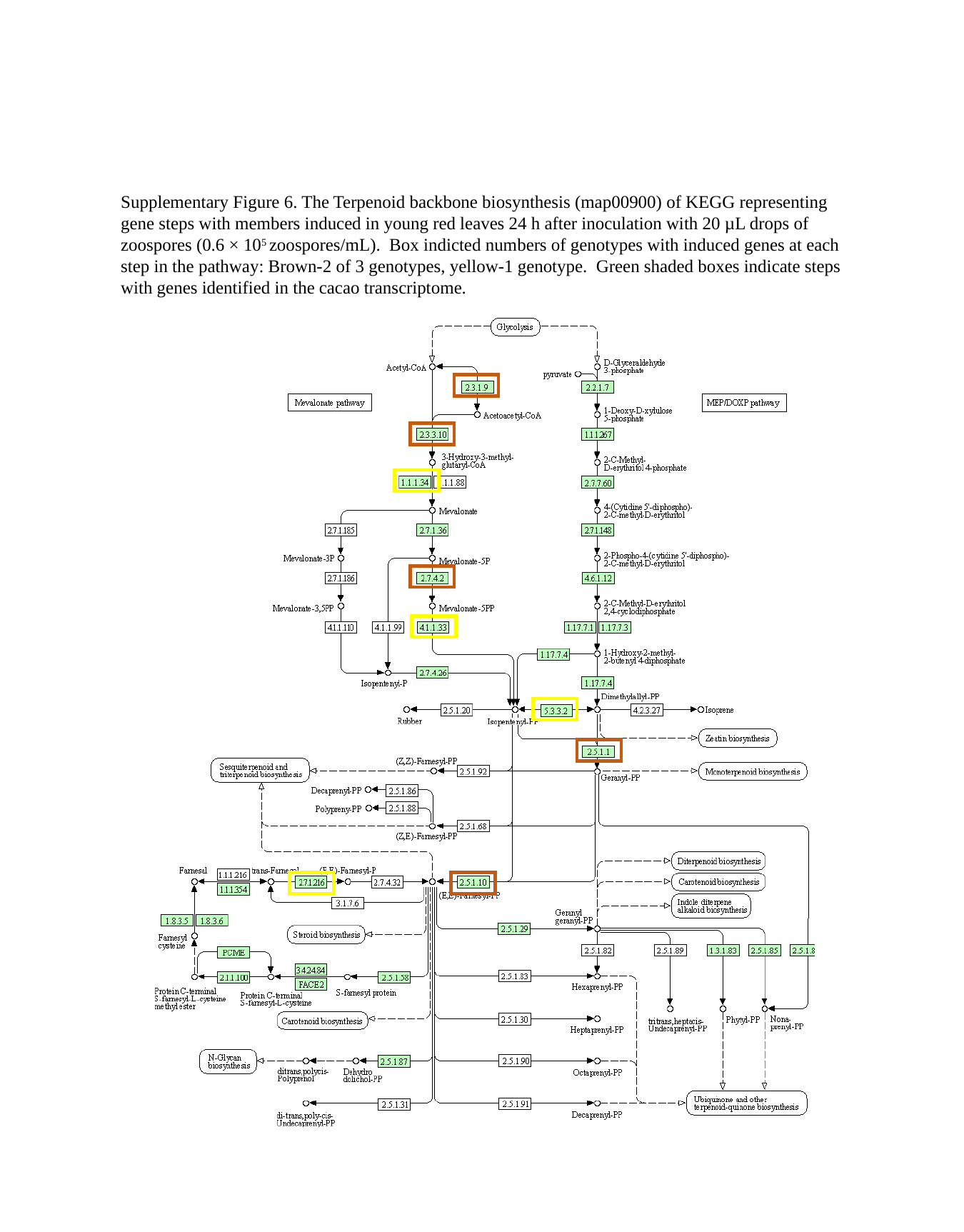

Supplementary Figure 6. The Terpenoid backbone biosynthesis (map00900) of KEGG representing gene steps with members induced in young red leaves 24 h after inoculation with 20 µL drops of zoospores (0.6 × 10^5^ zoospores/mL). Box indicted numbers of genotypes with induced genes at each step in the pathway: Brown-2 of 3 genotypes, yellow-1 genotype. Green shaded boxes indicate steps with genes identified in the cacao transcriptome.

## Slide 7
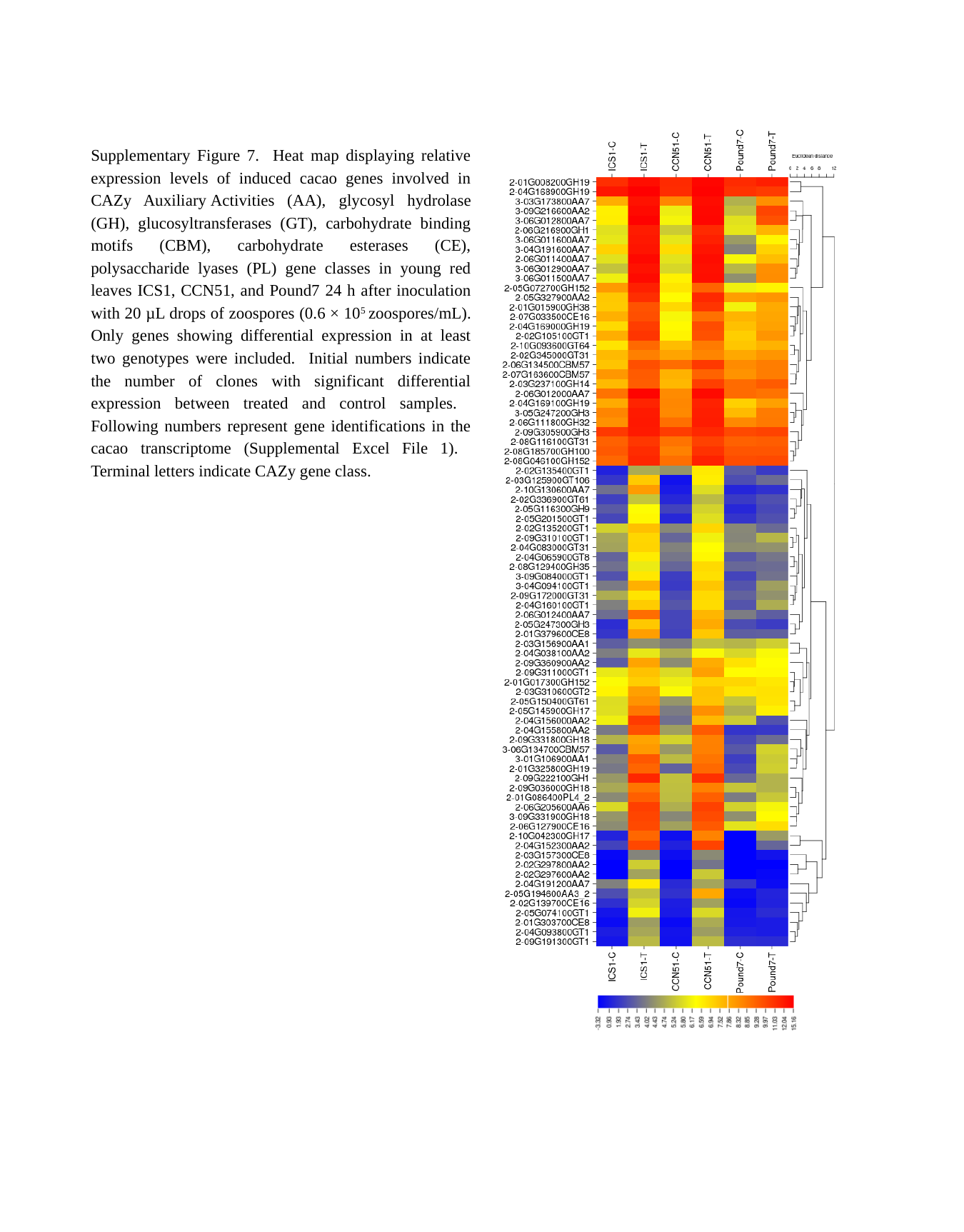

Supplementary Figure 7. Heat map displaying relative expression levels of induced cacao genes involved in CAZy Auxiliary Activities (AA), glycosyl hydrolase (GH), glucosyltransferases (GT), carbohydrate binding motifs (CBM), carbohydrate esterases (CE), polysaccharide lyases (PL) gene classes in young red leaves ICS1, CCN51, and Pound7 24 h after inoculation with 20 µL drops of zoospores (0.6 × 10^5^ zoospores/mL). Only genes showing differential expression in at least two genotypes were included. Initial numbers indicate the number of clones with significant differential expression between treated and control samples. Following numbers represent gene identifications in the cacao transcriptome (Supplemental Excel File 1). Terminal letters indicate CAZy gene class.

## Slide 8
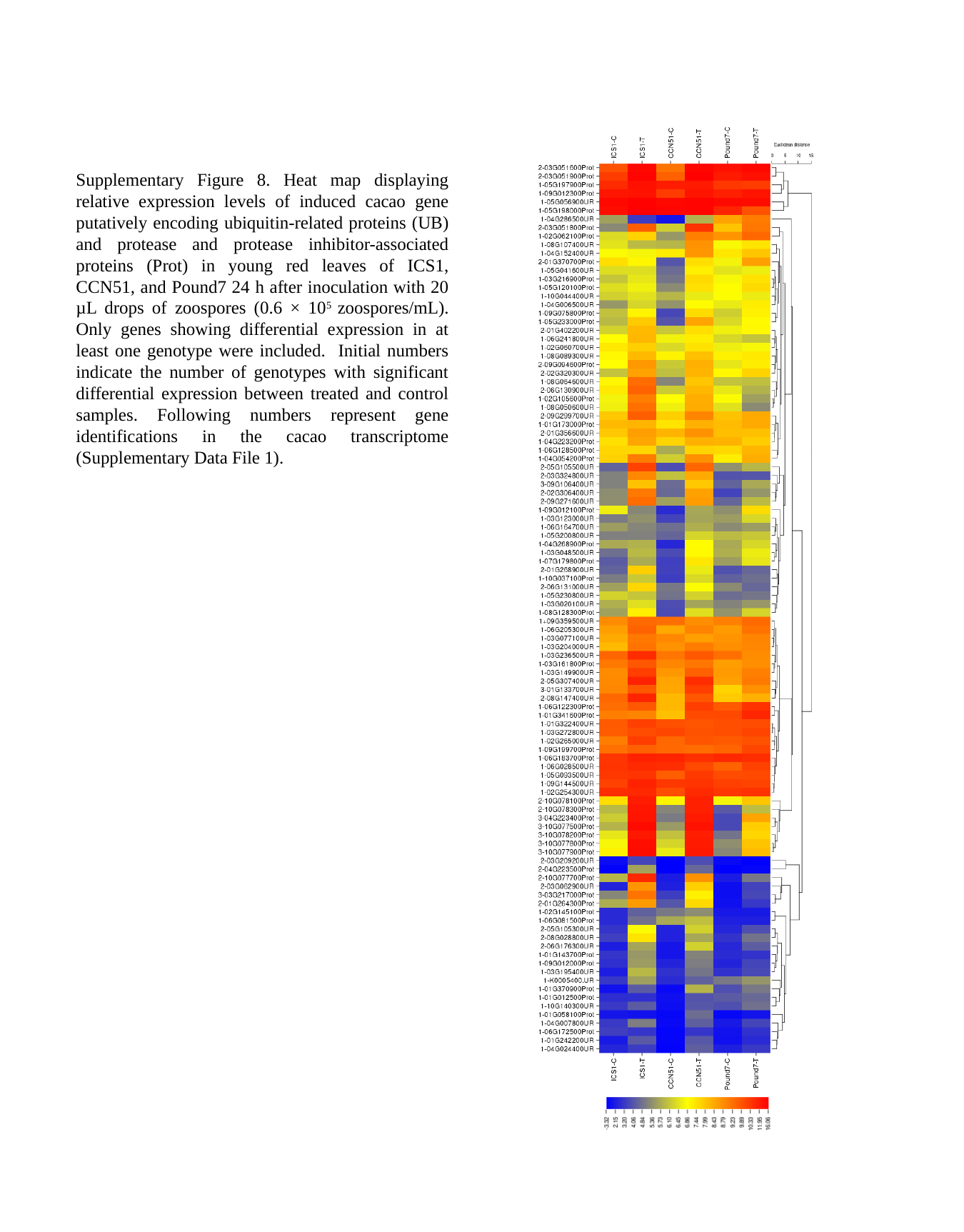

Supplementary Figure 8. Heat map displaying relative expression levels of induced cacao gene putatively encoding ubiquitin-related proteins (UB) and protease and protease inhibitor-associated proteins (Prot) in young red leaves of ICS1, CCN51, and Pound7 24 h after inoculation with 20 µL drops of zoospores (0.6 × 10^5^ zoospores/mL). Only genes showing differential expression in at least one genotype were included. Initial numbers indicate the number of genotypes with significant differential expression between treated and control samples. Following numbers represent gene identifications in the cacao transcriptome (Supplementary Data File 1).
